# Psychosocial stress and cannabinoid drugs affect acetylation of α-tubulin (K40) and gene expression in the prefrontal cortex of adult mice

**DOI:** 10.1371/journal.pone.0274352

**Published:** 2022-09-21

**Authors:** Jordi Tomas-Roig, Shyam Ramasamy, Diana Zbarsky, Ursula Havemann-Reinecke, Sigrid Hoyer-Fender

**Affiliations:** 1 Department of Psychiatry and Psychotherapy, University Medical Center Göttingen, Göttingen, Germany; 2 Center Nanoscale Microscopy and Molecular Physiology of the Brain (CNMPB), University of Göttingen, Göttingen, Germany; 3 Johann-Friedrich-Blumenbach-Institute of Zoology and Anthropology–Developmental Biology, GZMB, Georg-August-University Göttingen, Göttingen, Germany; University of Maryland at College Park, UNITED STATES

## Abstract

The dynamics of neuronal microtubules are essential for brain plasticity. Vesicular transport and synaptic transmission, additionally, requires acetylation of α-tubulin, and aberrant tubulin acetylation and neurobiological deficits are associated. Prolonged exposure to a stressor or consumption of drugs of abuse, like marihuana, lead to neurological changes and psychotic disorders. Here, we studied the effect of psychosocial stress and the administration of cannabinoid receptor type 1 drugs on α-tubulin acetylation in different brain regions of mice. We found significantly decreased tubulin acetylation in the prefrontal cortex in stressed mice. The impact of cannabinoid drugs on stress-induced microtubule disturbance was investigated by administration of the cannabinoid receptor agonist WIN55,212–2 and/or antagonist rimonabant. In both, control and stressed mice, the administration of WIN55,212–2 slightly increased the tubulin acetylation in the prefrontal cortex whereas administration of rimonabant acted antagonistically indicating a cannabinoid receptor type 1 mediated effect. The analysis of gene expression in the prefrontal cortex showed a consistent expression of *ApoE* attributable to either psychosocial stress or administration of the cannabinoid agonist. Additionally, *ApoE* expression inversely correlated with acetylated tubulin levels when comparing controls and stressed mice treated with WIN55,212–2 whereas rimonabant treatment showed the opposite.

## Introduction

Environmental factors such as traumatic events and consumption of drugs of abuse like cannabinoids can induce psychotic disorders as a result of neurobiological changes. These alterations comprise dysfunctions in neurogenesis, axonal growth, myelinization, synaptogenesis, synaptic pruning, and neuroendocrine regulation [1]. For instance, preclinical studies indicated that chronic exposure to psychosocial stress can cause neuroanatomical changes as e.g. inhibition of adult neurogenesis in the dentate gyrus, or dendritic atrophy in the hippocampus (HIPP) and the prefrontal cortex (PFC) [2–5]. Furthermore, chronic exposure to stress compromised the stability and function of axonal microtubules (MTs) promoting the hyperphosphorylation of the microtubule-binding protein Tau, and the formation of neurofibrillary tangles [6, 7]. Cytoskeletal alterations have also been reported in several neuropsychiatric diseases such as schizophrenia, major depressive disorder, and bipolar disorder. A common hallmark underlying neuropsychiatric disorders comprises an aberrant expression of tubulin isoforms and tubulin acetylation [8, 9].

MTs are assembled from stable heterodimers of α/β-tubulin and are constantly remodeled. In neuronal cells, MTs are important for compartmentation, rigidity, long-distance transport, and synaptic transmission [10]. Although they are highly conserved in evolution, MTs adapt to diverse cellular functions through microtubule-associated proteins (MAPs), posttranslational modifications of tubulins (PTMs), and binding proteins for specific PTMs. Thus, posttranslational modifications generate a ‘tubulin code’ that specifies the assignment and coordination of the complex functions of MTs [11–13]. An important modification is the acetylation of α-tubulin at lysine 40 (α-tubulin K40ac) that has been generally associated with MT stability. The addition of acetyl groups does not alter the MT ultrastructure but it is required for vesicular transport [14, 15]. The major tubulin acetyltransferase in mammals is the α-tubulin acetyltransferase 1 (ATAT1) causing complete loss of tubulin acetylation when deleted in mice. Although the loss of ATAT1 is not life-threatening, rodents underwent an enlargement of the forebrain lateral ventricles pointing out an important biological function of the *Atat1*-gene in this brain area [16–18]. Deacetylation of α-tubulin at K40ac is achieved by the histone deacetylase 6 (HDAC6) and the sirtuin type 2 (SIRT2), which, additionally, deacetylate further substrates [19, 20]. In the rodent brain, the main α-tubulin deacetylase is HDAC6 [21]. Additionally, HDAC6 recruits polyubiquitinated proteins to aggresomes and controls the fusion of autophagosomes and lysosomes [22–24]. HDAC6 is, therefore, involved in the clearance of misfolded proteins and protein aggregates [25, 26]. Furthermore, HDAC6 activity has been associated with emotional behavior like activity, anxiety, and depression [27, 28]. Thus, MTs in general and HDAC6, in particular, came into focus as promising therapeutic targets for neuropsychiatric disorders [29, 30]. To this end, HDAC inhibitors are emerging as potential antidepressant drugs [31].

Under physiological conditions, synaptic transmission and tissue homeostasis are biological mechanisms regulated by the cannabinoid receptor type-1 through G protein-coupled receptors (GPCRs) [32]. The cannabinoid receptor type-1 (CB1) is highly expressed in the brain and, specifically, found in axons and presynaptic terminals [33]. Upon stress exposure, CB1 and its endogenous ligands compromise the proper function of the brain [34]. CB1 mediates the pharmacological actions of synthetic cannabinoid drugs such as the full cannabinoid agonist WIN55,212–2 (referred to as W) and the inverse agonist rimonabant (referred to as R) [35]. Activation of CB1 by endogenous or exogenous cannabinoids regulates neuronal metabolism by decreasing mitochondrial cAMP and PKA [36]. Under pathological conditions of prolonged exposure to a stressor, the expression of CB1 is compromised [32, 35]. Socially defeat mice display a variety of molecular and physiological changes, commonly reported in psychiatric disorders, including changes in the endocannabinoid system and deregulation of β-actin contributing to dendritic and synaptic dysfunctions [7, 37, 38].

Here, we used a long-term psychosocial defeat protocol as a stress model for its etiological, predictive, discriminative, and face validity [39]. In our model, mice were exposed to daily psychosocial stress and finally acutely treated with the full cannabinoid agonist W and/or the inverse agonist R. We hypothesized that MTs might be affected in stressed animals, and thus, treatment with cannabinoid drugs could confer neuroprotection by remodeling the MT system. For this purpose, we evaluated stressed animals treated with cannabinoid drugs by quantifying α-tubulin acetylation and assessing differential gene expression. Among the wide spectrum of cerebral regions closely involved in stress-related disorders (for review see Ref. [40]), we directed our investigation on the PFC, a brain structure critically involved in social behaviour [41, 42].

## Materials and methods

### Ethics statement

All procedures were approved by the Göttingen University Institutional Animal Care and Use Committee and were in accordance with NIH guidelines for the use of animals in research and the European Communities Council Directive (2010/63/EU). The study was designed and carried out in compliance with the ARRIVE guidelines.

### Psychosocial stress experiment

Male mice of strains C57Bl6/J and FVB/N were purchased from Charles River Laboratories (Sulzfeld, Germany) and maintained under standard conditions (12-hours light/dark cycle with 6:00/18:00 lights on/off, a room temperature of 21±2°C, and food and water ad libitum). Altogether 120 male C57Bl6/J mice, aged 7–8 weeks, were used for the experiment. They were first habituated for one week and then half of them were subjected to the resident-intruder paradigm, while the other half were left undisturbed as the control group. One-year-old male FVB/N mice were kept individually and used as residents. For the social stress procedure, an intruder (C57Bl6/J mouse) was placed in the home cage of a resident (FVB/N mouse) where they freely interacted until the first aggression took place. Afterward, the intruder was isolated within the resident’s cage to prevent any further physical aggression but still subjected to odorous, visual, and vibrissae contact with the resident. The psychosocial stress protocol was performed one hour a day for 21 days. Control mice were placed in an empty cage once per day as their stressed counterparts did but without contact with FVB/N strain [34].

### Drug treatments

The CB1 receptor agonist WIN55,212–2 (referred to in the text as W, Sigma-Aldrich, St. Louis, USA) and the selective cannabinoid CB1 inverse agonist rimonabant (referred to in the text as R, Sequoia Research Products Ltd., Pangbourne, UK) were dissolved in a vehicle solution consisting of 10% DMSO (Sigma-Aldrich, St. Louis, USA) and 0.1% Tween-80 (Sigma-Aldrich, St. Louis, USA) in 0.9% saline. On day 21 of the experiment, the animals were injected intraperitoneally with a volume of 200 μl of drug and/or vehicle and then evaluated by behavioral testing [34]. The drugs W and R were administered at a concentration of 3mg/kg. Control and stressed animals were further divided into four subgroups of fifteen mice each, which received different treatments. Mice were treated twice with vehicle (V+V) as the control group, or subjected first to the vehicle and then W (V+W), or treated first with R and then with W (R+W), or injected first with R and then with vehicle (R+V).

### Sample preparation

Animals were sacrificed immediately after finishing the experiment. All mice were deeply anesthetized by intraperitoneal injection of 2,2,2-tribromethanol (Sigma-Aldrich, St. Louis, USA) followed by transcardial perfusion with cold 0.1% phosphate-buffered saline (PBS). Brain samples were isolated and frozen in liquid nitrogen. For the Western blot analyses, the brain samples from four randomly selected animals from each subgroup were chosen, giving a total of 32 animals. On the day of testing, tissues were homogenized in RIPA buffer containing protease inhibitors (Roche Applied Science, Penzberg, Germany). SDS-containing reducing sample buffer was added giving a final concentration of 1x [43]. Probes were denatured for 5 min at 60°C.

### Immuno-blotting

Protein lysates were separated on 10% SDS-polyacrylamide gels and transferred onto nitrocellulose membranes (Amersham Hybond-ECL, GE Healthcare) [43, 44]. The membrane was blocked in 5% dry milk in TBST (10 mM Tris-HCl pH 7.6, 150 mM NaCl, 0.05% Tween20) for one hour followed by incubation with the primary antibodies. These were mouse anti-acetylated α-tubulin (clone 6-11B-1; Santa Cruz, #sc-23950), and rabbit anti-ß-actin (Proteintech, #20536-1-AP). Both antibodies were resuspended in blocking solution and incubated with the blots at 4°C overnight by constant agitation in roller tubes. Blots were washed for 30 minutes in TBST followed by incubation with fluorophore-labelled secondary antibodies goat anti-mouse IRDye800CW (LI-COR Biosciences, #925–32210, lot #C81106-01), and goat anti-rabbit IRDye680RD (LI-COR Biosciences, #925–68071, lot #C80911-11) for 45 minutes at room temperature in roller tubes. After 45 minutes of washing in TBST, fluorescent images were captured by Odyssey CLx Imaging System (LI-COR Biosciences, Nebraska, USA), and proteins were quantified using the software Image Studio^TM^Lite (LI-COR Biosciences, Nebraska, USA).

### Protein quantification

For each individual Western blot experiment, all protein samples from one brain region, including the four different biological replicates for each treatment, were loaded onto four separate gels and immune-blotting and image capture were performed concurrently, using identical antibody dilutions, and identical image capture settings. The amounts of acetylated tubulin and ß-actin were measured in each lane, and the relative amount of acetylated tubulin in each lane was determined by calculating the ratio of acetylated tubulin to ß-actin in the same lane. The fold change of relative tubulin acetylation was determined using the average of the relative tubulin acetylation of the four biological control probes (obtained from mice not subjected to psychosocial stress, and injected with vehicle only, CTR (V+V)) as the reference, which was set as 1. The fold changes were calculated for all probes investigated concurrently on a set of four blots. These immune-blotting experiments and their analyses were repeated up to five times, always loading all probes onto a set of four gels as described above. Each run of Western blots was analyzed separately. Thus, up to five technical repetitions were used for each biological replicate. The average values of the relative amount of acetylated-α-tubulin of all technical replicates for each of the biological replicates were calculated and used for statistical analyses. Four biological replicates, i.e. samples from four mice, were analysed for each treatment, except for the HIPP probes from stressed mice treated with W+V, for which protein lysates from only three biological replicates could be quantified.

### Focused gene signature profiling

The prognostic 35-gene profile was performed in the digital transcript counting assay (nCounter-NanoString). The nCounter® technology permits the counting of individual nucleic acid molecules using digital detection of the fluorescent molecular barcodes attached to the target RNA. The mRNA hybridization, detection, and scanning were performed following the protocol provided by NanoString Technologies. 200–400 ng of RNA was taken as the starting material according to the manufacturer’s guidelines. Data were adjusted by scaling with the geometric mean of built-in control gene probes after log transformation (base 2) for each sample. The target genes were chosen according to the following criteria: genes involved in cytoskeleton architecture, neuropsychiatric disorders, and CNS myelination. The NanoStringnCounter™ code set was assigned as follows: *β-actin* (NM_007393.3), *apolipoprotein E* (*ApoE*; NM_009696.3), *C-X-C motif chemokine 12* (*CxCl12-Ɣ*; NM_001012477.2), *calreticulin* (*Calr*; NM_007591.3), *cannabinoid receptor 1* (*Cnr1*; NM_007726.3), *cannabinoid receptor 2* (*Cnr2*; NM_009924.3), *cub and sushi multiple domains 1* (*Csmd1*; NM_053171.2), *2′*,*3′-cyclic nucleotide 3′-phosphodiesterase* (*Cnp*; NM_001146318.1), *diacylglycerol lipase* (*Dagl-α*; NM_198114.2), *discoidin domain receptor 1* (*Ddr1*; NM_001198831.1), *dopamine receptor D1* (*Drd1*; NM_010076.3), *dopamine receptor D2* (*Drd2*; NM_010077.2), *dopamine receptor D3* (*Drd3*; NM_007877.1), *dopamine receptor D4* (*Drd4*; NM_007878.2), *dopamine receptor D5* (*Drd5*; NM_013503.2), *dystrobrevin binding protein 1* (*Dtnbp1*; NM_025772.4), *galactosylceramidase* (*Galc*; NM_008079.3), *glyceraldehyde-3-phosphate dehydrogenase* (*Gapdh*; NM_008084.2), *low density lipoprotein receptor* (*Ldlr*; NM_001252658.1), *myelin basic protein* (*Mbp*; NM_001025251.2), *myelin-associated glycoprotein (Mag*; NM_010758.2), *myelin oligodendrocyte glycoprotein* (*Mog*; NM_010814.2), *N-acyl phosphatidylethanolamine-specific phospholipase D* (*Nape-pld*; NM_178728.5), *nerve growth factor inducible* (*Vgf*; NM_001039385.1), *neuregulin 1* (*Nrg1*; NM_178591.2), *oligodendrocyte transcription factor 1* (*Olig1*; NM_016968.4), *oligodendrocyte transcription factor 2* (*Olig2*; NM_016967.2), *reticulon 4 receptor* (*Rtn4r*; NM_022982.2), *retinoid X receptor alpha* (*Rxra*; NM_011305.3), *ryanodine receptor 3* (*Ryr3*; NM_177652.2), *ski proto-oncogene* (*Ski*; NM_011385.2), *special AT-rich sequence-binding protein-2* (*Satb2*; NM_139146.2), *SRY-box 10* (*Sox10*; NM_011437.1*)*, *zinc finger protein 488* (*Zfp488*; NM_001013777.2), and *zinc finger protein GLI1* (*Gli1*; NM_010296.2). The mean value of the expression levels of *Gapdh* and *β-actin* was used as the standard control. Four mice per subgroup were used.

### Statistical analysis

Pairwise comparisons between the controls and stressed animals and between specific drug treatment and the vehicle were evaluated by Student’s t-test (unpaired, two-tailed). For the t-test, the average values of the fold-change values of acetylated-α-tubulin of all technical replicates of each biological replicate were used (S1 Table). Excel was used to verify normal distribution of the data. A two-way ANOVA was used to determine the effects of long-term stress and drug treatment on the expression of acetylated tubulin. The mean differences among the levels of one factor were determined by one-way ANOVA. Pairwise comparisons were performed using Bonferroni post hoc test. Gene expression profile was determined as a two-tailed *t*-test on the log-transformed normalized data that assumed unequal variance using nSolver™ 4.0. The distribution of the *t*-statistic was calculated using the Welch-Satterthwaite equation for the degrees of freedom in the estimation of the 95% confidence limits for observed differential expression between the two groups. Statistics and graphs were shown using GraphPad Prism 9.1.0. The resulting p-values were adjusted according to Benjamini-Hochberg [45]. The significance was set at p ≤ 0.05. In all figures and text, data are represented as mean ± SE.

## Results

### Psychosocial stress affects tubulin acetylation in the brain

**Fig 1:**
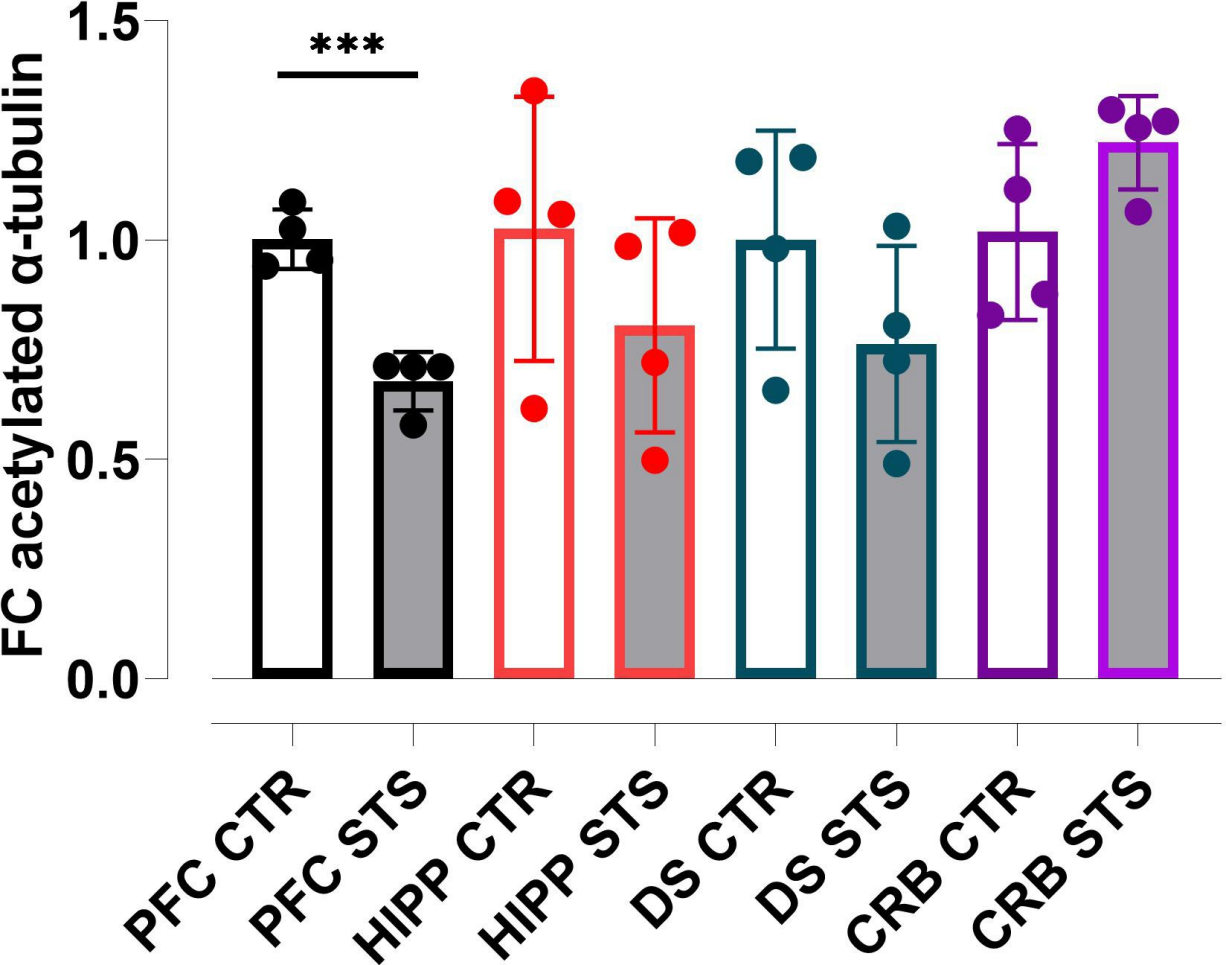
Psychosocial stress affects α-tubulin acetylation at K40 in different brain regions. Individual brain regions of control and stressed animals exposed to V+V were dissected and protein lysates prepared. Four biological replicates each were loaded onto SDS-gels, blotted, and finally tested with antibodies against acetylated-α-tubulin (K40) and β-actin. The relative amount of acetylated-α-tubulin in each lane was calculated by normalization for β-actin in the same lane. The fold changes of the relative amount of acetylated-α-tubulin in distinct brain regions of stressed animals were calculated using the mean value of the relative amount of acetylated-α-tubulin in the same brain region of control animals as reference. The dots represent the average values of the relative amount of acetylated-α-tubulin of all technical replicates for each of the four biological replicates. Light grey bars represent the stress group. CTR, control; STS, stress; V, vehicle; PFC, prefrontal cortex; HIPP, hippocampus; DS, dorsal striatum; CRB, cerebellum. One, two or three symbols indicate p < 0.05; p < 0.01, p<0.001, respectively.

We have previously reported that daily psychosocial stress affects body weight, exacerbates scratching activity, and increases the frequency of urination [34]. We asked here, whether the cytoskeleton was affected by repeated psychosocial stress. To this end, we investigated α-tubulin acetylation at K40 in different brain regions of stressed (STS) mice and control (CTR) mice by quantitative immune-blotting. In each experimental setting four biological replicates, i.e., four individual male mice were investigated, and every probe was loaded several times. The protein lysates of all biological replicates were treated and analysed simultaneously. Acetylated α-tubulin (K40) and ß-actin were indirectly detected by fluorophore-labelled secondary antibodies, and the relative quantities of acetylated α-tubulin and their fold-change differences were calculated as described in the materials and methods section. Fold changes of all technical and biological replicates were combined in Fig 1 (CTR *vs* STS, all brain regions). We observed a decrease in α-tubulin acetylation in stressed animals compared to the control animals in the PFC, the HIPP, and the dorsal striatum (DS), whereas an opposite pattern was observed in the cerebellum (CRB). The changes in α-tubulin acetylation were statistically significant for the PFC (t(34) = 3.903; p<0.000485***). No significant differences were found for the DS (t(35) = 2.702; p = 0.2037), HIPP (t(37) = 1.696; p = 0.29900) and CRB (t(29) = 1.662; p = 0.12295). Subsequently, our data indicate that psychosocial stress affects the acetylation of α-tubulin in the PFC. Since the PFC is critically involved in social behavior [41, 42], we aimed to further characterize this brain structure by assessing which genes were differentially expressed when comparing stressed and control mice subjected to different pharmacological interventions.

### Administration of the cannabinoid receptor agonist promotes tubulin acetylation in the prefrontal cortex

**Fig 2:**
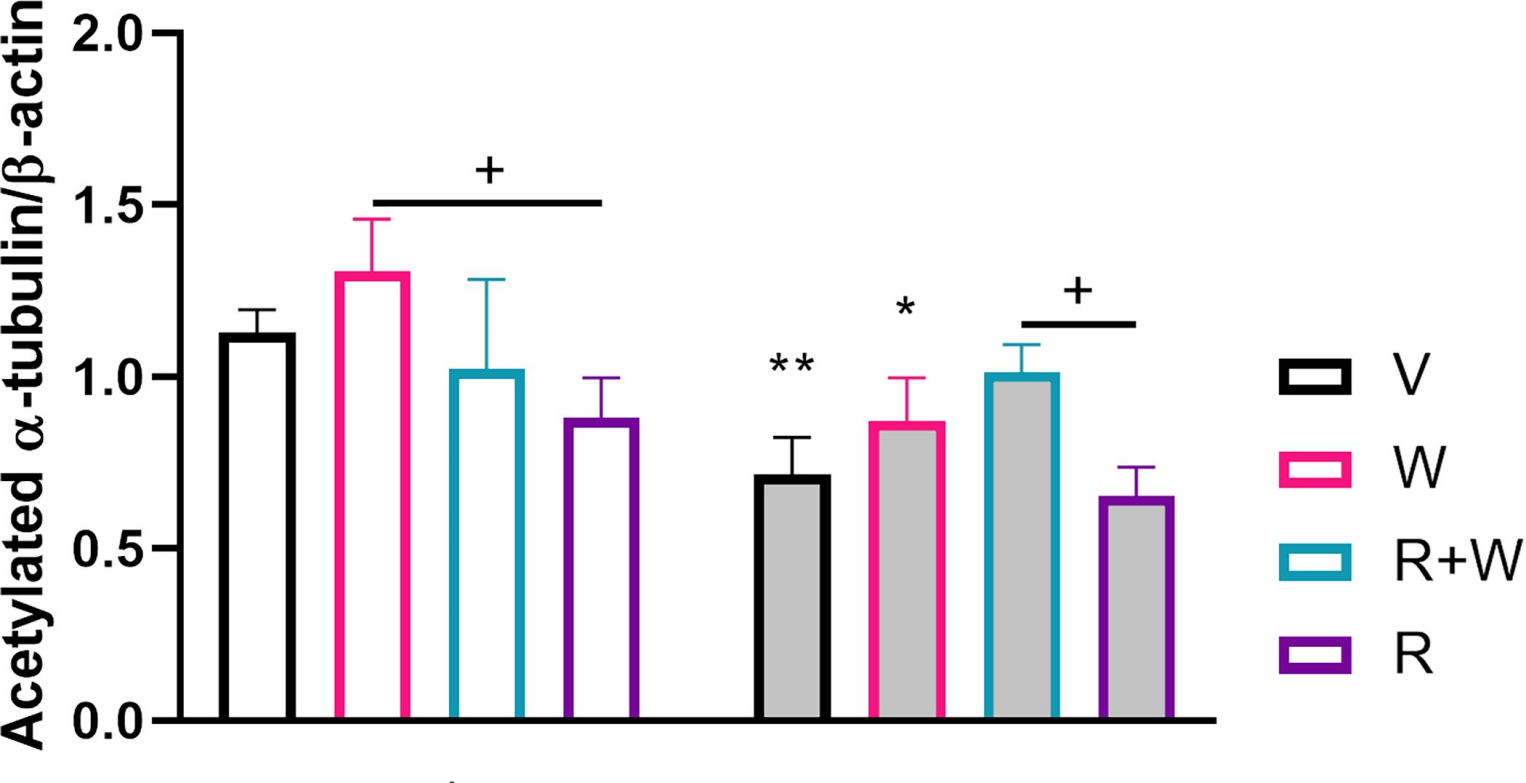
Effect of drug treatments on tubulin acetylation at K40 in the prefrontal cortex of stressed and non-stressed animals. We found higher expression of acetylated tubulin in controls treated with V+V when compared to their stressed counterparts. The use of V+W in controls increased the expression of acetylated tubulin in contrast to the control group treated with R+V and also did so when both drugs were administered simultaneously under stress conditions in comparison with those subjected to stress and treated with R+V. An * indicates significant differences between controls exposed to cannabinoid drugs and their respective V+V control group. Intragroup comparisons between drug‐treated mice are indicated by an underlined +. Otherwise underlined * pointed out significant comparisons between stressed mice and their matched counterparts. CTR, control; STS, stress; V, vehicle; W, WIN55212.2; R, rimonabant. One, two or three symbols indicate P < 0.05; P < 0.01, P<0.001, respectively.

Since our results demonstrated an effect of psychosocial stress on acetylation of tubulin in the brain, which presumably influence the tubulin dynamics and the organisation of the cytoskeleton, its stability and function, we wondered whether cannabinoid treatments might be effective in overcoming the stress-induced impact on MTs and neural function. We focused here on the PFC due to its particular importance for social behaviour and intellectual abilities, along with the highly significant decrease of tubulin acetylation in stressed animals (Fig 1). The two way ANOVA revealed a significant effect of stress (F(1, 29) = 29.35; p<0.001***), drug treatment (F(3, 29) = 7.56; p = 0.001***) as well as a significant interaction (stress x drug) (F(3, 29) = 3.91; p = 0.022*). The interaction of stress and drug treatment was evaluated by multiple comparisons post hoc tests (Table 1). Bonferroni post hoc test revealed higher expression of acetylated tubulin in controls treated with either vehicle or the CB1 agonist when compared to their stressed counterparts (p = 0.0091**; p = 0.021*, respectively) (Table 1; Fig 2). The use of cannabinoid receptor agonist in controls increased the expression of acetylated tubulin in contrast to the control group treated with the antagonist (p = 0.014*) (Table 1; Fig 2) and also did so when both drugs were administered simultaneously under stress conditions in comparison with those subjected to stress and treated with the cannabinoid antagonist (p = 0.033*) (Table 1; Fig 2). Thus, our results indicate that treatment with the CB1 agonist protects from stress-induced tubulin deacetylation while the administration of the antagonist favours the removal of acetyl groups in the PFC.

**Table 1:** Acetylated α-tubulin profile in the prefrontal cortex of control and stressed mice upon vehicle or cannabinoid drug exposure.

	**Equality of variances**		**One-way ANOVA**				
	**(Levene test)**						
					**Pairwise comparisons**	**(M**_**i**_ **-M**_**j**_ **) + SD**	**p value**
Acetylated tubulin	W(7,22) = 0.99	n.s.	F (7,22) = 8.84	***<0.001	CTR V+V vs STS V+V	0.41+0.01	.009
Expression					CTR V+W vs STS V+W	0.43+0.11	.021
					CTR V+W vs CTR R+V	0.42+0.10	.014
					STS R+V vs STS R+W	-0.35+0.09	.033

Column titles from left to right: Levene’s test; One-way ANOVA; Pairwise comparisons; (Mi-Mj) + SD; p-value. CTR, control; STS, stress; V, vehicle; W, WIN55,212–2; R, rimonabant; (Mi-Mj) + SD, average difference between 2 distinct treatments including the standard deviation.

### Acetylation of α-tubulin in the hippocampus, the dorsal striatum, and the cerebellum after administration of cannabinoid drugs

**Fig 3:**
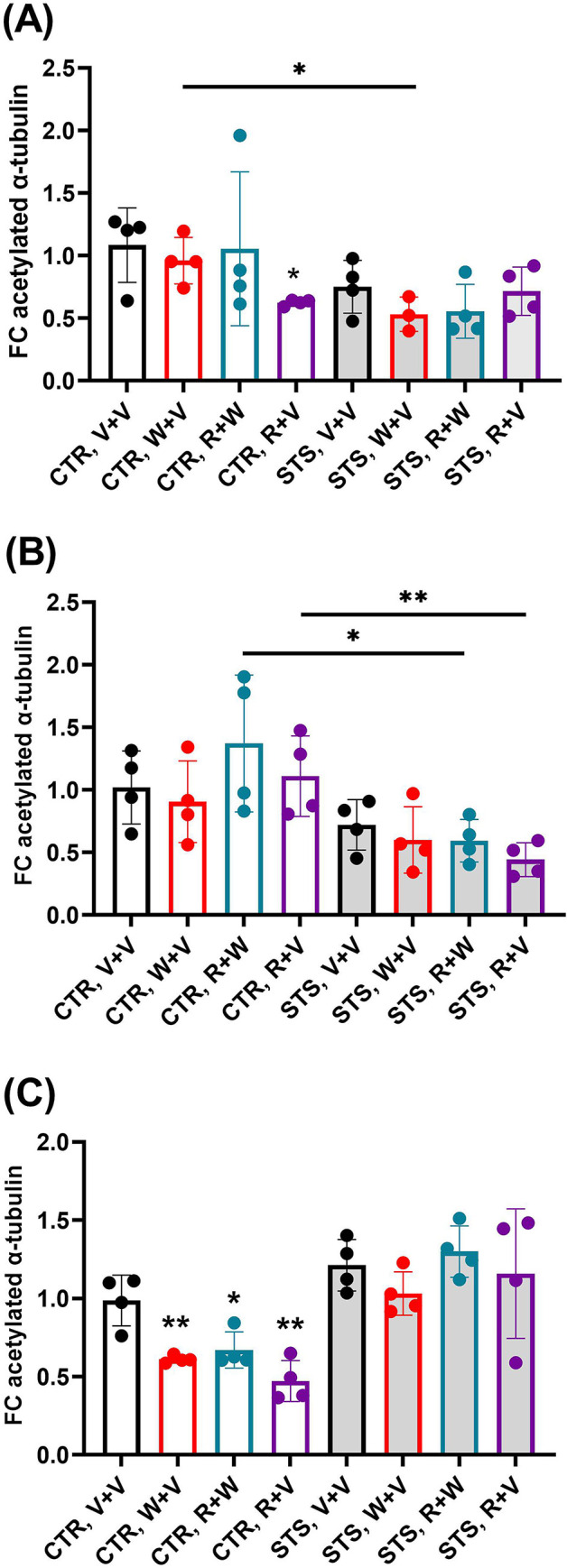
Psychosocial stress and cannabinoid drugs alter acetylation of α-tubulin at K40 in distinct brain regions. (A) We found a significant reduction of tubulin acetylation in the hippocampus from control animals following R+V treatment, and in stressed animals following W+V treatment compared to their matched controls (CTR, V+V and CTR, W+V, respectively). No significant changes were observed in stressed animals compared to controls or in stressed animals treated with drugs. The dots represent the average values of the relative amount of acetylated-α-tubulin of all technical replicates for each biological replicate. (B) In the dorsal striatum, significant changes in tubulin acetylation were observed in stressed animals treated with either R+V or R+W when compared to their matched control groups. The dots represent the average values of the relative amount of acetylated-α-tubulin of all technical replicates for each of the four biological replicates. (C) In the cerebellum, the administration of cannabinoid drugs in controls caused a reduction in tubulin acetylation in comparison to controls subjected to V+V. The dots represent the average values of the relative amount of acetylated-α-tubulin of all technical replicates for each of the four biological replicates. Light grey bars represent the stress group. An * indicates significant differences between controls exposed to cannabinoid drugs and their respective V+V control group. Otherwise underlined * pointed out significant comparisons between stressed mice and their matched counterparts. CTR, control; STS, stress; V, vehicle; W, WIN55,212–2; R, rimonabant. One, two or three symbols indicate P < 0.05; P < 0.01, P<0.001, respectively.

We also investigated α-tubulin acetylation in the HIPP, the DS, and the CRB after administration of either the cannabinoid receptor 1 agonist W, the inverse agonist R, or both cannabinoid drugs in both stressed and control animals. In the HIPP of control animals, administration of R+V caused a decrease in tubulin acetylation (t(26) = 3.577; p = 0.021577*) whereas neither V+W nor R+W resulted in a significant change in comparison to the control group treated with vehicle alone (Fig 3, panel A). In stressed animals, a significant change in tubulin acetylation was not observed compared to controls subjected to V+V, nor by drug administration. Administration of W+V in stressed animals caused a significant decrease in tubulin acetylation when compared with their matched controls (CTR, W+V, p = 0.020951*) (Fig 3, panel A).

In the DS, administration of either V+W, R+V, or R+W did not alter tubulin acetylation status in either the control or stress group. Significant changes in tubulin acetylation were observed in stressed animals treated with R+V or both drugs (R+W) when compared to their matched control group (p = 0.008861** and p = 0.034778*, respectively) (Fig 3, panel B).

In the CRB of control animals, the administration of either V+W, R+W, or R+V induced a significant decrease in tubulin acetylation as compared to the vehicle-treated controls (CTR, V+V *vs* V+W: t(24) = 3.102; p = 0.003811**; V+V *vs* R+W: t(23) = 2.896; p = 0.019340*; V+V *vs* R+V: t(23) = 3.793; p = 0.002640**) (Fig 3, panel C). Stressed animals treated with vehicle did not display significantly different tubulin acetylation than their matched controls. Furthermore, the administration of either V+W, R+V, or both cannabinoid drugs did not change tubulin acetylation in stressed animals compared to socially-defeat animals subjected to vehicle alone (Fig 3, panel C).

To conclude, the effect on tubulin acetylation by either long-term exposure to stress or administration of cannabinoid drugs was different according to the brain region but especially prominent in the PFC. The effects of stress are brain-region-specific as pointed out previously [46].

### Gene expression signature in the prefrontal cortex

We reported changes in gene expression in the PFC when socially defeat mice were subjected to cannabinoid drugs by use of the digital transcript counting (nCounter) assay (NanoString) [35]. The target genes were chosen according to the following criteria: genes involved in cytoskeleton architecture, neuropsychiatric disorders, and CNS myelination. Long term exposure to psychosocial stress increased the expression of *ApoE* (t(6) = 6.61; p_adj_ = 0.01**), *CxCl12ɣ* (t(6) = 6.47; p_adj_ = 0.02*), *Dtnbp1* (t(6) = 5.85; p_adj_ = 0.02*), *Ski* (t(6) = 5.16; p_adj_ = 0.04*), and *Cnr1* (t(6) = 4.71; p_adj_ = 0.05*) when compared to their matched controls (Table 2). Non-stressed animals treated simultaneously with both drugs underwent a remarkable downregulation of *ApoE* (t(6) = -7.92; p_adj_<0.001***) and *Rtn4r* (t(6) = -9.12; p_adj_<0.001***) in comparison with their controls (Table 2). The administration of R+W in non-stressed mice resulted in lower levels of *ApoE* (t(6) = 11.07; p_adj_<0.001***), *Rtn4r* (t(6) = 11.77; p_adj_<0.001***), *Vgf* (t(6) = 7.4; p_adj_ = 0.01**), *Rxra* (t(6) = 6.92; p_adj_ = 0.01**), *Cnp* (t(6) = 5.6; p_adj_ = 0.02*), *Mbp* (t(6) = 4.72; p_adj_ = 0.05*), and Cnr1 (t(6) = 6.64; p_adj_ = 0.03*) than the control group treated with V+W (Table 2). Daily exposure to psychosocial stress induced a decrease in *Mag* expression upon V+W administration (t(6) = 5.73; p_adj_ = 0.04*) in contrast to those exposed to vehicle alone (Table 2). Social defeat mice treated with R+V displayed lower expression of *ApoE* (t(6) = -8.96; p_adj_ = 0.02*) than those treated with V+W (Table 2). Stressed mice treated with the cannabinoid agonist displayed an upregulation of *ApoE* (t(6) = 6.48; p_adj_ = 0.05*) when compared to their non-stressed counterparts (Table 2) Socially defeat mice subjected to R+W showed elevated levels of *ApoE* (t(6) = 9.18; p_adj_<0.001***), *Cnp* (t(6) = 7.22; p_adj_<0.001***), *Vgf* (t(6) = 8.35; p_adj_<0.001***), *Drd1* (t(6) = 7.81; p_adj_<0.001***), *Drd5* (t(6) = 5.95; p_adj_ = 0.02*), *Rxra* (t(6) = 7.2; p_adj_<0.001***), *Zfp488* (t(6) = 10.34; p_adj_<0.001***), *Mbp* (t(6) = 6.92; p_adj_ = 0.01**), *Cnr1* (t(6) = 6.51; p_adj_ = 0.02*), and *Ryr3* (t(6) = 5.55; p_adj_ = 0.03*) in comparison to their counterparts non-exposed to stress (Table 2).

**Table 2:** Gene expression signature in the prefrontal cortex derived from control and stressed mice upon vehicle or cannabinoid drug exposure.

Comparison	Gene symbol	Accession	Fold change	q-value
STS V+V *vs* CTR V+V	*ApoE*	NM_009696.3	1.44	.01
	*CxCl12ɣ*	NM_001012477.2	1.54	.02
	*Dtnbp1*	NM_025772.4	1.36	.02
	*Ski*	NM_011385.2	1.46	.04
	*Cnr1*	NM_007726.3	1.63	.05
CTR R+W *vs* CTR V+V	*ApoE*	NM_009696.3	0.55	< .001
	*Rtn4r*	NM_022982.2	0.43	< .001
CTR R+W *vs* CTR V+W	*ApoE*	NM_009696.3	0.45	< .001
	*Rtn4r*	NM_022982.2	0.37	< .001
	*Vgf*	NM_001039385.1	0.46	.01
	*Rxra*	NM_011305.3	0.31	.01
	*Cnp*	NM_001146318.1	0.38	.02
	*Cnr1*	NM_007726.3	0.49	.03
	*Mbp*	NM_001025251.2	0.32	.05
STS V+W *vs* STS V+V	*Mag*	NM_010758.2	0.47	.04
STS R+V *vs* STS V+W	*ApoE*	NM_009696.3	0.80	.02
STS V+W *vs* CTR V+W	*ApoE*	NM_009696.3	1.40	.05
STS R+W *vs* CTR R+W	*ApoE*	NM_009696.3	2.23	.001
	*Cnp*	NM_001146318.1	3.03	.001
	*Vgf*	NM_001039385.1	2.44	.001
	*Drd1*	NM_010076.3	8.34	.001
	*Rxra*	NM_011305.3	3.28	.001
	*Zfp488*	NM_001013777.2	2.90	.001
	*Mbp*	NM_001025251.2	3.90	.01
	*Drd5*	NM_013503.2	4.18	.02
	*Cnr1*	NM_007726.3	2.07	.02
	*Ryr3*	NM_177652.2	4.76	.03

Column titles from left to right: Comparison; Gene symbol; Accession; Fold Change; q-value. N = 4 mice per subgroup. CTR, control; STS, stress; V, vehicle; W, WIN55,212–2; R, rimonabant; q-value, adjusted p-value.

### Pearson’s chi-squared correlation analysis between gene expression and tubulin acetylation in the PFC

**Fig 4:**
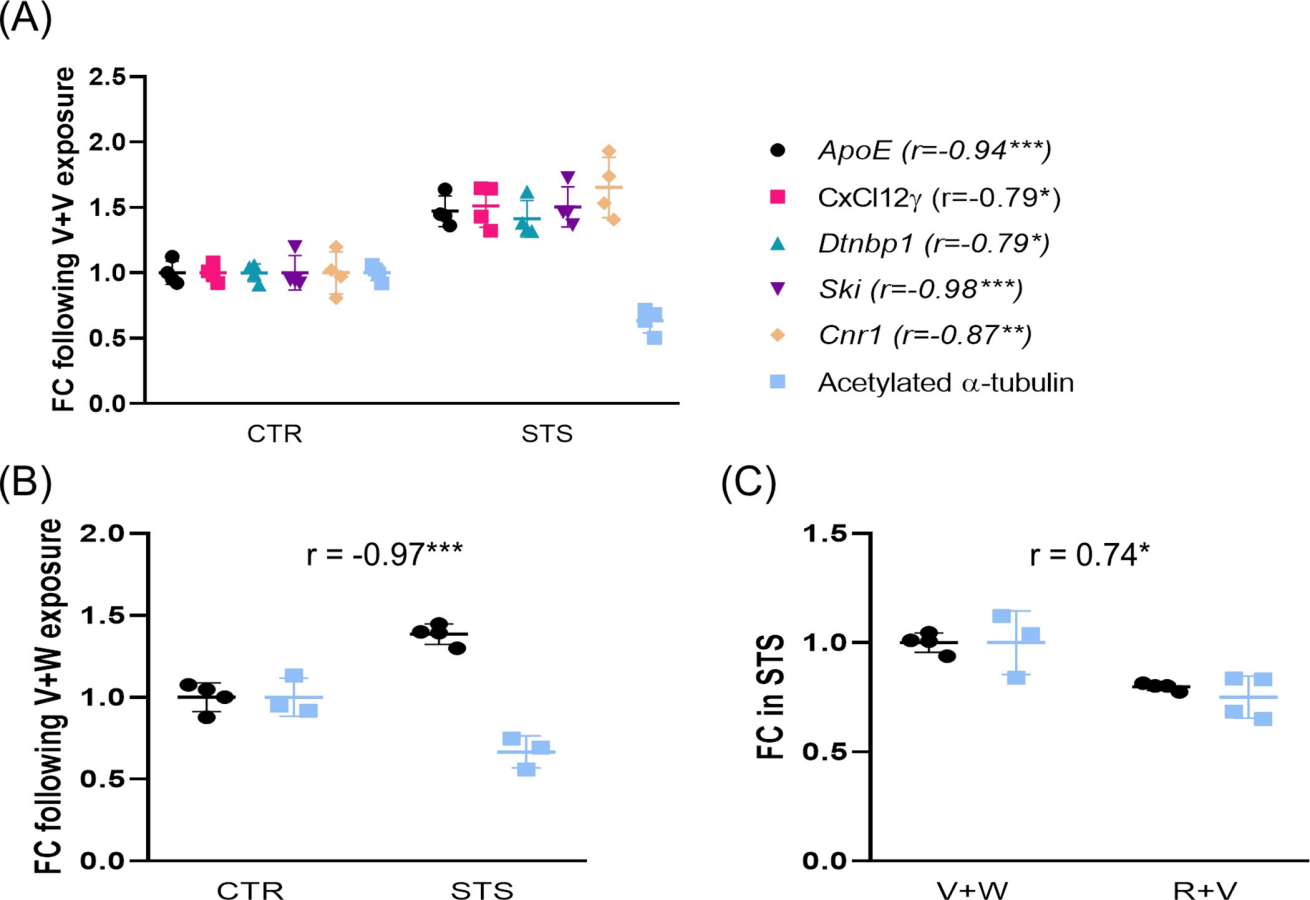
Correlation analysis between acetylated α-tubulin at K40 and gene expression in the prefrontal cortex. We reported an inverse association between acetylated α-tubulin levels and either *ApoE*, *CxCl12ɣ*, *Dtnbp1*, *Ski*, or *Cnr1* when compared controls and defeat mice subjected to V+V (panel A); and also did the expression of *ApoE* upon V+W treatment when comparing the control and the stress group (panel B). A positive correlation between acetylated α-tubulin and *ApoE* was reported between V+W and R+V under stress conditions (panel C). CTR, control; STS, stress; V, vehicle; W, WIN55,212–2; R, rimonabant. One, two or three symbols indicate P < 0.05; P < 0.01, P<0.001, respectively.

Administration of drugs had a consistent effect on tubulin acetylation in both control and stressed animals when the PFC was examined (Fig 4). The administration of the inverse agonist led to a decrease in tubulin acetylation when compared to the control group treated with vehicle whereas the administration of either R+V or R+W reduced tubulin acetylation in contrast to what was observed upon W treatment. We, therefore, analyzed the correlation between tubulin acetylation and gene expression signature reporting an inverse association between acetylated tubulin levels and either *ApoE* (*r = -*0.94; p<0.001***), *CxCl12ɣ* (r = -0.79; p = 0.019*), *Dtnbp1* (r = -0.79; p = 0.019*), *Ski* (r = -0.98; p<0.001***) or *Cnr1* (r = -0.87; p = 0.005**) expression when comparing controls and defeat mice subjected to vehicle (Fig 4, panel A); and also did so, after acute injection with V+W when compared the control and the stress group (r = -0.97; p = 0.001***) (Fig 4, panel B). A positive correlation between acetylated tubulin and *ApoE* levels was reported following either V+W or R+V under repeated stress (r = 0.74; p = 0.05*) (Fig 4, panel C).

## Discussion

The dynamic rearrangement of the neuronal MTs is crucial for brain plasticity by enabling the remodelling of dendrites and axons [46]. Persistent stressful conditions revealed structural alterations of the cytoskeleton [6, 7]. We asked here, whether psychosocial stress affects the neuronal cytoskeleton by investigating the post-translational tubulin modification based on the addition of acetyl groups. We found a significant reduction of tubulin acetylation in the PFC, whereas non-significant differences were observed in the HIPP, the DS, and the CRB of mice exposed to chronic psychosocial stress. Reduced levels of tubulin acetylation in the PFC were accompanied by differential gene expression. Particularly, an upregulation of *ApoE*, *CxCl12γ*, *Dtnbp1*, *Ski*, and *Cnr1* in the PFC of stressed mice was determined. The administration of V+W promoted an increase of both *ApoE* and *Cnr1* levels in comparison to R+W. Upregulation of *ApoE* was also observed in stressed mice treated with V+W in contrast to their matched controls. The data highlighted a consistent expression of *ApoE* deregulated by stress and also by the administration of the CB1 agonist which might indicate a CB1 mediated effect. Therefore, we investigated whether the administration of cannabinoid drugs might have a potential therapeutic effect to overcome the stress-induced cytoskeletal modifications.

### The effect of psychosocial stress and the impact of cannabinoid drugs on neuronal microtubules

Neuronal remodelling and brain plasticity are regulated by the MT system. In animals, stressful conditions can impair neuronal plasticity. Stress induces neuronal atrophy in the HIPP by retraction of apical dendrites, cell death, and decreased neurogenesis [4, 47–49]. However, it is not restricted to this brain region. In particular, chronic restraint stress in experimental animals also provokes atrophy in the PFC [46, 50]. Since acetylation of tubulin contributes to cytoskeletal stability, a decrease might lead to synaptic and dendrite alterations [51]. Consistently, we found a decreased tubulin acetylation in the PFC derived from mice exposed to psychosocial stress.

Our results indicate an imbalance of tubulin acetylation and deacetylation in social defeat mice pointing to either reduced expression or inhibition of the enzymatic activity of the acetyltransferase *Atat1* or hyper-activity of the HDAC6 tubulin deacetylase. A change in gene expression, neither for *Atat1* nor *Hdac6*, was not assessed by Nanostring gene expression arrays. However, it is well known that ATAT1 is highly expressed in the cerebral cortex and the HIPP (retrieved from The Human Protein Atlas at https://www.proteinatlas.org/ENSG00000137343-ATAT1/tissue), whereas HDAC6 is highly expressed in the brain and especially in the cortex, the HIPP, and the CRB ([52]; retrieved from The Human Protein Atlas at https://www.proteinatlas.org/ENSG00000094631-HDAC6/tissue). Since HDAC6 is the main α-tubulin deacetylase in the brain, changes in tubulin acetylation are most likely due to the deregulation of HDAC6 activity. HDAC6, additionally, targets also the chaperone Hsp90 and the redox regulatory proteins peroxiredoxin I and II (Prx I and II), both of them involved in the stress response [21, 53, 54]. Perturbation of acetylated tubulin dynamics has been reported in neuropsychiatric diseases and stress-related disorders [8–10]. Furthermore, HDAC6 affects the emotional behaviour of experimental animals since *Hdac6*-deficient mice exhibit hyperactivity, less anxiety, and anti-depressant-like behaviour [27]. Cells respond to stress immediately with the formation of cytoplasmic stress granules by recruitment of HDAC6. Interestingly, an appropriate activity of HDAC6 is required to counteract oxidative stress [55]. Besides that, HDAC6 is essential for the clearance of misfolded proteins and protein aggregates that are degraded by either the ubiquitin-proteasome pathway or autophagy. Indeed, HDAC6 binds to polyubiquitinated misfolded proteins favouring their retrograde MT transport to aggresomes. Thus, HDAC6 is a component of aggresomes that increases the efficiency and selectivity of autophagic degradation and protects cells from stress response caused by aggregation of misfolded proteins [22, 56]. Subsequently, an increase of HDAC6 activity could promote autophagy and clearance of misfolded proteins and protein aggregates, e.g., aggregates of hyperphosphorylated Tau proteins generated by chronic stress [6]. A decrease in tubulin acetylation as observed in the PFC of the stress group could thus indicate a neuroprotective function attributable to the overactivation of HDAC6. Elevated levels of HDAC6 deacetylase activity are under the tight control of the kinases Aurora A and GSK3-ß [57, 58]. Activation of Aurora A is regulated by HEF1 (human enhancer of filamentation 1) that in turn is activated by HIF-1α (hypoxia-inducible factor 1α) [59]. Thus, putatively, stressful events might induce hypoxic conditions that favour Aurora A and HDAC6 activation and lastly, tubulin deacetylation. Blockage of GSK3-ß inhibits deacetylase activity and simultaneously increases tubulin acetylation. GSK3-ß is a substrate of the PI3K/AKT pathway and becomes phosphorylated and inactivated by stimulation of AKT. Thus, the inhibition of the AKT pathway activates HDAC6 reducing tubulin acetylation. Additionally, GSK3-ß negatively regulates mitochondrial and anterograde axonal transport [58, 60]. The activity of the PI3K/AKT pathway is controlled by upstream molecules such as the CXCL12-CXCR4 and DTNBP1. Drug-induced tubulin acetylation and MT stabilization are suppressed upon CXCL12 activation in prostate cancer cells [61]. Dysbindin-1 regulates synapse morphology, synaptic plasticity, vesicle trafficking, and reduced dopamine-induced phosphorylation of AKT/GSK3ß [62]. Thus, elevated levels *of CxCl12-ɣ* or *Dtnbp1*, as observed in the PFC of stressed animals, might contribute to HDAC6 hyperactivation and eventually tubulin deacetylation.

In the HIPP, the DS, and the CRB, activation of the G-protein coupled cannabinoid receptor CB1, by administration of Δ9-tetrahydrocannabinol (THC), increases phosphorylation of AKT, whereas the selective CB1 antagonist R blocked it [63]. Furthermore, the phosphorylation of GSK3-ß is also increased by THC administration [63]. We showed here, that the administration of the CB1 agonist slightly increased tubulin acetylation in the PFC. In contrast, a reduction was found in the CRB of the control group when the same drug was administered. As expected, activation of CB1 by use of the cannabinoid agonist activates AKT kinase that in turn inactivates GSK3-ß by phosphorylation eventually blocking the activity of HDAC6 resulting in elevated levels of tubulin acetylation. Such an effect was observed exclusively in the PFC, although not significantly. We reported higher expression of *Cnr1* in the PFC of stressed animals that could prevent the hyperactivation of HDAC6 via PI3K/AKT signaling. Instead, we observed a decrease in tubulin acetylation levels pointing out that the overexpression of *Cnr1* measured here might act as a compensatory mechanism to counteract the reduced levels of tubulin acetylation. However, further analyses warrant investigation. Collectively, our results demonstrate that distinct brain regions respond differentially to either prolonged stress or the administration of cannabinoid drugs. Furthermore, the different levels of tubulin acetylation reported here could suggest that neuroprotection mediated by CB1 is quite variable and cell type-specific. In summary, our findings revealed a reduction of tubulin acetylation under chronic stress when the PFC was studied. This fact could be an indicator of HDAC6 hyperactivity. Dysfunctions in tubulin acetylation under stress conditions might indicate that cellular architecture and vesicle transport are compromised [14, 15]. But taking into account the protective role of HDAC6 against cellular stress, an alternative scenario could suggest that changes in tubulin acetylation could confer protection against chronic stress instead of being just a detrimental outcome.

### Long-term effects of psychosocial stress on gene expression in the prefrontal cortex

Chronic psychosocial stress, such as the social defeat model, causes a variety of molecular, physiological, and behavioral changes [34, 35, 39]. Upon repeated psychosocial stress, differential gene expression is found in the CRB and other brain regions [35, 39]. Elevated levels of *ApoE*, *CxCl12ɣ*, *Dtnbp1*, *Ski*, *and Cnr* were found in stressed mice subjected to V+V when compared to their matched controls (Table 2). As reported, most of these genes are related to neuronal functioning. ApoE is involved in lipid metabolism and acts as the principal cholesterol carrier in the brain [64]. Experimental and clinical studies both revealed that *ApoE* levels are related to stress response [65, 66]. Furthermore, repeated psychosocial stress per se stimulates inflammatory response by activating leukocyte extravasation through the blood-brain barrier into the brain [67]. This process is tightly regulated by the complex *Cxcl12-Cxcr4*. Repeated social defeat promotes the synthesis of *CxCl12* within the brain exacerbating the pro-inflammatory reaction [68] in line with the results reported here. *Dtnbp1* is involved in distinct biological processes, including dendritic spines and synapse formation [69]. Overexpression of *Dtnbp1* under stress conditions might confer vulnerability to psychotropic drugs [70]. *Ski* is a transcriptional modulator required for the expansion of precursor cells in the neuroepithelium or skeletal muscle lineages [71]. *Ski* has been involved in distinct biological processes such as muscle homeostasis, axonal growth, myelination, hematopoietic cell differentiation, regulation of T-cells as well as in many complex pathologies [72]. However, its function in the context of prolonged stress remains to be elucidated and warrants further investigation.

### Effects of cannabinoid drugs under non-stress conditions

The administration of both drugs reduced *ApoE* and *Rtn4r* levels when compared to those subjected to vehicle alone. Here, we demonstrated that alterations in the endocannabinoid signalling by either long-term stress or acute cannabinoid treatment are accompanied by changes in *ApoE* expression [35, 73]. We reported elevated levels of *ApoE* in the control group after V+W injection in contrast to those treated with R+W. Similarly, Russell and colleagues (2010) found an increase of *ApoE* attributable to the drug W [74] while the antagonist acted oppositely [75]. *Rtn4r* encodes a glycosylphosphatidylinositol-anchored protein remarkably present in the PFC [76] that has been involved in oligodendrocyte proliferation [77], cytoskeleton organization [78], and neuronal processes such as neurotransmission, regeneration, sprouting, and plasticity [79]. A decrease in *Rtn4r* following R+W treatment was measured when compared to vehicle alone which might act as a compensatory mechanism against functional disruption of the neural PFC system. Indeed, elevated levels of *Rtn4r* compromise the proper function of PFC [80]. Acute administration of the cannabinoid agonist in non-stressed mice led to an overexpression of *Cnp* and *Mbp* when compared to those treated with R+W. CNP participates in RNA splicing, trafficking, and metabolism in mature oligodendrocytes [81–84]. In contrast, MBP regulates the adhesion of compact multilayered myelin sheath [85]. An increase in both myelin-related genes might indicate deficits in myelin CNS architecture [80]. Levels of *Vgf*, encoding a neuropeptide involved in energy balance, were higher following V+W treatment [86], while the inverse agonist had the opposite effect [87]. This is in line with the results reported upon V+W administration in comparison to R+W. Interestingly, elevated levels of this neuropeptide exacerbates the inflammatory response in rodents [88]. Retinoic acid receptor RXR-alpha (*Rxra*) participates in the regulation of calcium signalling [89] and synapse formation [90]. Upon activation, RXRA stimulates oligodendrocyte differentiation [91] and promotes the phagocytic functions of microglia [89]. The data presented herein show elevated levels of *Rxra* following treatment with the CB1 agonist in contrast to R+W. We speculate that this might indicate dysfunctions in oligodendrocyte differentiation, synapse formation, and immune response. The expression of CB1 receptor, encoded by the *Cnr1* gene, was higher following V+W than when both drugs were administered in agreement with [80].

Socially defeat mice treated with V+W exhibited lower expression of *Mag* in comparison to those subjected to vehicle alone. The overall differences observed between these groups could be attributable to the dosage of the cannabinoid agonist applied. An *in vitro* model of oligodendrocyte differentiation revealed that a low dosage of the cannabinoid agonist confers neuroprotection while the administration of higher doses of the drug aggravates demyelination [92]. Loss of *Mag*, encoding a transmembrane glycoprotein localized at peri-axonal regions of oligodendroglia, is associated with oligodendrocyte dysfunctions [93]. Under stress conditions, intraperitoneal injection of the cannabinoid antagonist induced a reduction in *ApoE* levels in comparison to V+W. In contrast, stressed mice subjected to the cannabinoid agonist displayed higher expression of *ApoE* than their non-stressed counterparts which is in keeping with [74]. Socially defeat mice treated with both cannabinoid drugs underwent elevated levels of *ApoE*, *Cnp*, *Vgf*, *Drd1*, *Drd5*, *Rxra*, *Zfp488*, *Mbp*, *Cnr1*, and *Ryr3* in comparison to their non-stressed counterparts (Table 2). Mice subjected to chronic stress and acutely treated with both cannabinoid drugs showed elevated levels of *ApoE* [73–75]. Co-administration of both drugs under stress resulted in higher levels of *Vgf* which, in turn, could be attributable to the cannabinoid agonist W [86]. Indeed, exposure to either psychosocial stress or acute administration of the inverse agonist acted in the opposite direction than the cannabinoid agonist did [87, 94]. Dopaminergic neurotransmission is essential for cerebral function, controlling various physiological mechanisms including cognition, locomotion, neuroendocrine activity, emotional and motivational aspects [95]. Recent studies have demonstrated that midbrain dopaminergic neurons are particularly vulnerable to microtubule disruptions [96]. We measured a prominent expression of *Drd5* in stressed mice subjected to R+W which could be explained by a stress-mediated effect [97] rather than the consumption of cannabinoid drugs [98]. The administration of R+W under the influence of stress resulted in more *Rxra* expression than in their non-stressed counterparts. Elevated levels of *Rxra* might be associated with the use of cannabinoid drugs [35, 99]. There are no data available as yet on the role of *Rxra* in the context of chronic stress. Subsequently, the function of *Rxra* under repeated psychosocial stress warrants further investigation. The proliferation of oligodendrocyte precursor cells into myelinating oligodendrocytes is regulated by distinct transcription factors like ZFP488 [100]. We reported an increase of *Zfp488* when socially defeat mice were subjected to both cannabinoids in comparison to their non-stressed counterparts [101]. This fact might indicate dysfunctions in oligodendrocyte differentiation [100]. Upon coadministration with R+W, stressed mice displayed higher *Mbp* expression than their control counterparts indicating perturbations in CNS myelination [85]. These alterations could be explained by the administration of synthetic cannabinoid drugs [80] rather than exposure to a repeated stressor [102]. The expression of *Cnr1* was higher in stressed mice subjected to R+W than their controls subjected to equal pharmacological treatment. Such increase could be attributed to either drug treatment [103] or exposure to chronic stress [104]. Ca^2+^ release into the cytoplasm of neurons is regulated by ryanodine receptors [105]. Changes in RYR3 activity lead to an imbalance in intracellular levels of calcium contributing to an impairment in neurotransmission [106] and lastly neurodegeneration [107]. We reported higher expression of *Ryr3* in social defeat mice treated with R+W than their matched non-stressed mice. This evidence might be explained by either chronic exposure to a stressor [108] or acute cannabinoid administration [109].

## Conclusions

In summary, we concluded that the decrease in acetylated MTs most likely affects neurotransmission, which nevertheless could promote neuroprotection under long-term stress conditions. Additionally, enhanced HDAC6 activity attributable to lower levels of tubulin acetylation could promote autophagy and clearance of misfolded proteins and aggregates in stressed animals [6]. In this regard, we can speculate that an increase of *ApoE* under the influence of stress might contribute to neuroprotection as well. It has been shown that *ApoE* participates in MT polymerization and neurite extension [110, 111]. HDAC6 affects both the nuclear localization of ApoE and the microtubule-organizing center [112]. Thus, elevated levels of ApoE together with a decrease in tubulin acetylation might indicate a dynamic reassembling of the MTs. Collectively, the overexpression of *ApoE*, *CxCl12-ɣ*, *Dtnbp1*, and *Cnr1* in the PFC of stressed animals may trigger MT destabilization while tubulin deacetylation could promote MT reorganization acting as a protective mechanism against the side effects of stress. We found a consistent expression of *ApoE* attributable to either psychosocial stress [65, 66] or administration of the CB1 agonist [74]. Interestingly, the expression of ApoE was inversely correlated with acetylated tubulin levels when comparing controls and stressed mice subjected to the CB1 agonist whereas the use of the CB1 inverse agonist acted oppositely. This fact might indicate a CB1 receptor-mediated effect. The diverse effects on tubulin acetylation observed in the studied brain regions of both control and stressed animals with or without pharmacological treatment might indicate that HDAC6 activity is differently regulated in the brain acting simultaneously on different cellular mechanisms to safeguard homeostatic processes for the proper function of the CNS.

## Supporting information

S1 Fig Exemplary of Western blots. Detection of acetylated-α-tubulin (55 kDa) and β-actin (40 kDa) by green fluorescence and red fluorescence spectrum, respectively. Protein lysates from the prefrontal cortex of four biological replicates derived from the control (CTR; 17–32) and the stress group (STS; 33–44) were used. Control animals (17–32): 17–20 (V +V group), 21–24 (V+W group), 25–28 (R+W group), 29–32 (R+V group). Stressed animals (33–44): 33–36 (V+V group), 37–40 (V+W group), 41–44 (R+W group), 45–48 (R+V group). M = molecular mass marker. CTR, control; STS, stress.
(TIF)

S1 Table Analyses of relative α-tubulin acetylation in the prefrontal cortex (PFC), the hippocampus (HIPP), the dorsal striatum (DS), and the cerebellum (CRB) in control (CTR) and stressed (STS) mice. Treatment with either vehicle **(**V+V), rimonabant (R+V), WIN55,212–2 (W+V), or rimonabant and WIN55,212–2 (R+W).
(XLSX)

S1 File (DOCX)

## References

[1] van Os J, Kenis G, Rutten BPF. The environment and schizophrenia. Nature. 2010;468:203–12. doi: 10.1038/nature09563 21068828

[2] Uno H, Tarara R, Else JG, Suleman MA, Sapolsky RM. Hippocampal damage associated with prolonged and fatal stress in primates. J Neurosci. 1989 May;9(5):1705–11. doi: 10.1523/JNEUROSCI.09-05-01705.1989 2723746 PMC6569823

[3] Gould E, Tanapat P, McEwen BS, Flügge G, Fuchs E. Proliferation of granule cell precursors in the dentate gyrus of adult monkeys is diminished by stress. Proc Natl Acad Sci U S A. 1998 Mar;95(6):3168–71. doi: 10.1073/pnas.95.6.3168 9501234 PMC19713

[4] Magariños AM, Verdugo JMG, McEwen BS. Chronic stress alters synaptic terminal structure in hippocampus. Proc Natl Acad Sci [Internet]. 1997 Dec 9;94(25):14002 LP– 14008. Available from: http://www.pnas.org/content/94/25/14002.abstract doi: 10.1073/pnas.94.25.14002 9391142 PMC28422

[5] Radley JJ, Arias CM, Sawchenko PE. Regional differentiation of the medial prefrontal cortex in regulating adaptive responses to acute emotional stress. J Neurosci. 2006 Dec;26(50):12967–76. doi: 10.1523/JNEUROSCI.4297-06.2006 17167086 PMC6674963

[6] Lopes S, Vaz-Silva J, Pinto V, Dalla C, Kokras N, Bedenk B, et al. Tau protein is essential for stress-induced brain pathology. Proc Natl Acad Sci U S A. 2016 Jun;113(26):E3755–63. doi: 10.1073/pnas.1600953113 27274066 PMC4932951

[7] McEwen BS, Bowles NP, Gray JD, Hill MN, Hunter RG, Karatsoreos IN, et al. Mechanisms of stress in the brain. Nat Neurosci. 2015 Oct;18(10):1353–63. doi: 10.1038/nn.4086 26404710 PMC4933289

[8] Wong GT-H, Chang RC-C, Law AC-K. A breach in the scaffold: the possible role of cytoskeleton dysfunction in the pathogenesis of major depression. Ageing Res Rev. 2013 Jan;12(1):67–75. doi: 10.1016/j.arr.2012.08.004 22995339

[9] Marchisella F, Coffey ET, Hollos P. Microtubule and microtubule associated protein anomalies in psychiatric disease. Cytoskeleton (Hoboken). 2016 Oct;73(10):596–611. doi: 10.1002/cm.21300 27112918

[10] Kapitein LC, Hoogenraad CC. Which way to go? Cytoskeletal organization and polarized transport in neurons. Mol Cell Neurosci. 2011 Jan;46(1):9–20. doi: 10.1016/j.mcn.2010.08.015 20817096

[11] Verhey KJ, Gaertig J. The tubulin code. Cell Cycle. 2007 Sep;6(17):2152–60. doi: 10.4161/cc.6.17.4633 17786050

[12] Garnham CP, Roll-Mecak A. The chemical complexity of cellular microtubules: tubulin post-translational modification enzymes and their roles in tuning microtubule functions. Cytoskeleton (Hoboken). 2012 Jul;69(7):442–63. doi: 10.1002/cm.21027 22422711 PMC3459347

[13] Janke C. The tubulin code: molecular components, readout mechanisms, and functions. J Cell Biol. 2014 Aug;206(4):461–72. doi: 10.1083/jcb.201406055 25135932 PMC4137062

[14] Howes SC, Alushin GM, Shida T, Nachury M V, Nogales E. Effects of tubulin acetylation and tubulin acetyltransferase binding on microtubule structure. Mol Biol Cell. 2014 Jan;25(2):257–66. doi: 10.1091/mbc.E13-07-0387 24227885 PMC3890346

[15] Even A, Morelli G, Broix L, Scaramuzzino C, Turchetto S, Gladwyn-Ng I, et al. ATAT1-enriched vesicles promote microtubule acetylation via axonal transport. Sci Adv. 2019 Dec;5(12):eaax2705. doi: 10.1126/sciadv.aax2705 31897425 PMC6920029

[16] Kalebic N, Sorrentino S, Perlas E, Bolasco G, Martinez C, Heppenstall PA. αTAT1 is the major α-tubulin acetyltransferase in mice. Nat Commun. 2013;4:1962. doi: 10.1038/ncomms2962 23748901

[17] Kim G-W, Li L, Ghorbani M, You L, Yang X-J. Mice lacking α-tubulin acetyltransferase 1 are viable but display α-tubulin acetylation deficiency and dentate gyrus distortion. J Biol Chem. 2013 Jul;288(28):20334–50. doi: 10.1074/jbc.M113.464792 23720746 PMC3711300

[18] Li L, Jayabal S, Ghorbani M, Legault L-M, McGraw S, Watt AJ, et al. ATAT1 regulates forebrain development and stress-induced tubulin hyperacetylation. Cell Mol Life Sci. 2019 Sep;76(18):3621–40. doi: 10.1007/s00018-019-03088-3 30953095 PMC11105686

[19] Hubbert C, Guardiola A, Shao R, Kawaguchi Y, Ito A, Nixon A, et al. HDAC6 is a microtubule-associated deacetylase. Nature. 2002 May;417(6887):455–8. doi: 10.1038/417455a 12024216

[20] North BJ, Marshall BL, Borra MT, Denu JM, Verdin E. The human Sir2 ortholog, SIRT2, is an NAD+-dependent tubulin deacetylase. Mol Cell. 2003 Feb;11(2):437–44. doi: 10.1016/s1097-2765(03)00038-8 12620231

[21] Bobrowska A, Donmez G, Weiss A, Guarente L, Bates G. SIRT2 ablation has no effect on tubulin acetylation in brain, cholesterol biosynthesis or the progression of Huntington’s disease phenotypes in vivo. PLoS One. 2012;7(4):e34805. doi: 10.1371/journal.pone.0034805 22511966 PMC3325254

[22] Kawaguchi Y, Kovacs JJ, McLaurin A, Vance JM, Ito A, Yao TP. The deacetylase HDAC6 regulates aggresome formation and cell viability in response to misfolded protein stress. Cell. 2003 Dec;115(6):727–38. doi: 10.1016/s0092-8674(03)00939-5 14675537

[23] Hook SS, Orian A, Cowley SM, Eisenman RN. Histone deacetylase 6 binds polyubiquitin through its zinc finger (PAZ domain) and copurifies with deubiquitinating enzymes. Proc Natl Acad Sci U S A. 2002 Oct;99(21):13425–30. doi: 10.1073/pnas.172511699 12354939 PMC129689

[24] Lee J-Y, Koga H, Kawaguchi Y, Tang W, Wong E, Gao Y-S, et al. HDAC6 controls autophagosome maturation essential for ubiquitin-selective quality-control autophagy. EMBO J. 2010 Mar;29(5):969–80. doi: 10.1038/emboj.2009.405 20075865 PMC2837169

[25] Yan J. Interplay between HDAC6 and its interacting partners: essential roles in the aggresome-autophagy pathway and neurodegenerative diseases. DNA Cell Biol. 2014 Sep;33(9):567–80. doi: 10.1089/dna.2013.2300 24932665

[26] Odagiri S, Tanji K, Mori F, Miki Y, Kakita A, Takahashi H, et al. Brain expression level and activity of HDAC6 protein in neurodegenerative dementia. Biochem Biophys Res Commun. 2013 Jan;430(1):394–9. doi: 10.1016/j.bbrc.2012.11.034 23159615

[27] Fukada M, Hanai A, Nakayama A, Suzuki T, Miyata N, Rodriguiz RM, et al. Loss of deacetylation activity of Hdac6 affects emotional behavior in mice. PLoS One. 2012;7(2):e30924. doi: 10.1371/journal.pone.0030924 22328923 PMC3273475

[28] Liu R, Dang W, Du Y, Zhou Q, Jiao K, Liu Z. SIRT2 is involved in the modulation of depressive behaviors. Sci Rep. 2015 Feb;5:8415. doi: 10.1038/srep08415 25672834 PMC4325337

[29] Pandey UB, Nie Z, Batlevi Y, McCray BA, Ritson GP, Nedelsky NB, et al. HDAC6 rescues neurodegeneration and provides an essential link between autophagy and the UPS. Nature. 2007 Jun;447(7146):859–63. doi: 10.1038/nature05853 17568747

[30] Esteves AR, Palma AM, Gomes R, Santos D, Silva DF, Cardoso SM. Acetylation as a major determinant to microtubule-dependent autophagy: Relevance to Alzheimer’s and Parkinson disease pathology. Biochim Biophys acta Mol basis Dis. 2019 Aug;1865(8):2008–23. doi: 10.1016/j.bbadis.2018.11.014 30572013

[31] Fuchikami M, Yamamoto S, Morinobu S, Okada S, Yamawaki Y, Yamawaki S. The potential use of histone deacetylase inhibitors in the treatment of depression. Prog Neuropsychopharmacol Biol Psychiatry. 2016 Jan;64:320–4. doi: 10.1016/j.pnpbp.2015.03.010 25818247

[32] Turu G, Hunyady L. Signal transduction of the CB1 cannabinoid receptor. J Mol Endocrinol. 2010 Feb;44(2):75–85. doi: 10.1677/JME-08-0190 19620237

[33] Ladarre D, Roland AB, Biedzinski S, Ricobaraza A, Lenkei Z. Polarized cellular patterns of endocannabinoid production and detection shape cannabinoid signaling in neurons. Front Cell Neurosci. 2014;8:426. doi: 10.3389/fncel.2014.00426 25610369 PMC4285097

[34] Tomas-Roig J, Piscitelli F, Gil V, Quintana E, Ramió-Torrentà LL, del Río JA, et al. Effects of repeated long-term psychosocial stress and acute cannabinoid exposure on mouse corticostriatal circuitries: Implications for neuropsychiatric disorders. CNS Neurosci Ther. 2018;24(6). doi: 10.1111/cns.12810 29388323 PMC5969305

[35] Tomas-Roig J, Havemann-Reinecke U. Gene expression signature in brain regions exposed to long-term psychosocial stress following acute challenge with cannabinoid drugs. Psychoneuroendocrinology. 2019;102. doi: 10.1016/j.psyneuen.2018.11.023 30476795

[36] Bénard G, Massa F, Puente N, Lourenço J, Bellocchio L, Soria-Gómez E, et al. Mitochondrial CB₁ receptors regulate neuronal energy metabolism. Nat Neurosci. 2012 Mar;15(4):558–64. doi: 10.1038/nn.3053 22388959

[37] English JA, Dicker P, Föcking M, Dunn MJ, Cotter DR. 2-D DIGE analysis implicates cytoskeletal abnormalities in psychiatric disease. Proteomics. 2009 Jun;9(12):3368–82. doi: 10.1002/pmic.200900015 19562803

[38] Piubelli C, Carboni L, Becchi S, Mathé AA, Domenici E. Regulation of cytoskeleton machinery, neurogenesis and energy metabolism pathways in a rat gene-environment model of depression revealed by proteomic analysis. Neuroscience. 2011 Mar;176:349–80. doi: 10.1016/j.neuroscience.2010.12.043 21195137

[39] Tomas-Roig J, Piscitelli F, Gil V, Moore T, Agbemenyah H, Salinas-Riester G, et al. Social defeat leads to changes in the endocannabinoid system: An overexpression of calreticulin and motor impairment in mice. Behav Brain Res. 2016;303(1872–7549):34–43. doi: 10.1016/j.bbr.2016.01.036 26815100

[40] Duval ER, Javanbakht A, Liberzon I. Neural circuits in anxiety and stress disorders: A focused review. Vol. 11, Therapeutics and Clinical Risk Management. 2015. p. 115–26. doi: 10.2147/TCRM.S48528 25670901 PMC4315464

[41] Blair KS, Geraci M, Otero M, Majestic C, Odenheimer S, Jacobs M, et al. Atypical modulation of medial prefrontal cortex to self-referential comments in generalized social phobia. Psychiatry Res—Neuroimaging. 2011;193(1):38–45. doi: 10.1016/j.pscychresns.2010.12.016 21601433 PMC3105197

[42] Sripada C, Angstadt M, Liberzon I, McCabe K, Phan KL. Aberrant reward center response to partner reputation during a social exchange game in generalized social phobia. Depress Anxiety. 2013;30(4):353–61. doi: 10.1002/da.22091 23576237 PMC3987865

[43] Laemmli UK. Cleavage of structural proteins during the assembly of the head of bacteriophage T4. Nature. 1970 Aug;227(5259):680–5. doi: 10.1038/227680a0 5432063

[44] Towbin H, Staehelin T, Gordon J. Electrophoretic transfer of proteins from polyacrylamide gels to nitrocellulose sheets: procedure and some applications. Proc Natl Acad Sci U S A. 1979 Sep;76(9):4350–4. doi: 10.1073/pnas.76.9.4350 388439 PMC411572

[45] Benjamini Y, Hochberg Y. Controlling the False Discovery Rate: A Practical and Powerful Approach to Multiple Testing. J R Stat Soc Ser B [Internet]. 1995;57(1):289–300. Available from: http://www.jstor.org/stable/2346101

[46] Pittenger C, Duman RS. Stress, depression, and neuroplasticity: a convergence of mechanisms. Neuropsychopharmacol Off Publ Am Coll Neuropsychopharmacol. 2008 Jan;33(1):88–109. 10.1038/sj.npp.1301574 17851537

[47] McEwen BS. Stress and hippocampal plasticity. Annu Rev Neurosci. 1999;22:105–22. doi: 10.1146/annurev.neuro.22.1.105 10202533

[48] Duman RS, Malberg J, Nakagawa S, D’Sa C. Neuronal plasticity and survival in mood disorders. Biol Psychiatry. 2000 Oct;48(8):732–9. doi: 10.1016/s0006-3223(00)00935-5 11063970

[49] Bianchi M, Heidbreder C, Crespi F. Cytoskeletal changes in the hippocampus following restraint stress: role of serotonin and microtubules. Synapse. 2003 Sep;49(3):188–94. doi: 10.1002/syn.10230 12774303

[50] Radley JJ, Rocher AB, Miller M, Janssen WGM, Liston C, Hof PR, et al. Repeated stress induces dendritic spine loss in the rat medial prefrontal cortex. Cereb Cortex. 2006 Mar;16(3):313–20. doi: 10.1093/cercor/bhi104 15901656

[51] Janke C, Montagnac G. Causes and Consequences of Microtubule Acetylation. Curr Biol. 2017 Dec;27(23):R1287–92. doi: 10.1016/j.cub.2017.10.044 29207274

[52] Broide RS, Redwine JM, Aftahi N, Young W, Bloom FE, Winrow CJ. Distribution of histone deacetylases 1–11 in the rat brain. J Mol Neurosci. 2007;31(1):47–58. doi: 10.1007/BF02686117 17416969

[53] Kovacs JJ, Murphy PJM, Gaillard S, Zhao X, Wu J-T, Nicchitta C V, et al. HDAC6 regulates Hsp90 acetylation and chaperone-dependent activation of glucocorticoid receptor. Mol Cell. 2005 May;18(5):601–7. doi: 10.1016/j.molcel.2005.04.021 15916966

[54] Parmigiani RB, Xu WS, Venta-Perez G, Erdjument-Bromage H, Yaneva M, Tempst P, et al. HDAC6 is a specific deacetylase of peroxiredoxins and is involved in redox regulation. Proc Natl Acad Sci U S A. 2008 Jul;105(28):9633–8. doi: 10.1073/pnas.0803749105 18606987 PMC2443817

[55] Kwon S, Zhang Y, Matthias P. The deacetylase HDAC6 is a novel critical component of stress granules involved in the stress response. Genes Dev. 2007 Dec;21(24):3381–94. doi: 10.1101/gad.461107 18079183 PMC2113037

[56] Iwata A, Riley BE, Johnston JA, Kopito RR. HDAC6 and microtubules are required for autophagic degradation of aggregated huntingtin. J Biol Chem. 2005 Dec;280(48):40282–92. doi: 10.1074/jbc.M508786200 16192271

[57] Pugacheva EN, Jablonski SA, Hartman TR, Henske EP, Golemis EA. HEF1-dependent Aurora A activation induces disassembly of the primary cilium. Cell. 2007 Jun;129(7):1351–63. doi: 10.1016/j.cell.2007.04.035 17604723 PMC2504417

[58] Chen S, Owens GC, Makarenkova H, Edelman DB. HDAC6 regulates mitochondrial transport in hippocampal neurons. PLoS One. 2010 May;5(5):e10848. doi: 10.1371/journal.pone.0010848 20520769 PMC2877100

[59] Kim S-H, Xia D, Kim S-W, Holla V, Menter DG, Dubois RN. Human enhancer of filamentation 1 Is a mediator of hypoxia-inducible factor-1alpha-mediated migration in colorectal carcinoma cells. Cancer Res. 2010 May;70(10):4054–63. doi: 10.1158/0008-5472.CAN-09-2110 20442290 PMC2871069

[60] Morfini G, Szebenyi G, Elluru R, Ratner N, Brady ST. Glycogen synthase kinase 3 phosphorylates kinesin light chains and negatively regulates kinesin-based motility. EMBO J. 2002 Feb;21(3):281–93. doi: 10.1093/emboj/21.3.281 11823421 PMC125832

[61] Bhardwaj A, Srivastava SK, Singh S, Arora S, Tyagi N, Andrews J, et al. CXCL12/CXCR4 signaling counteracts docetaxel-induced microtubule stabilization via p21-activated kinase 4-dependent activation of LIM domain kinase 1. Oncotarget. 2014 Nov;5(22):11490–500. doi: 10.18632/oncotarget.2571 25359780 PMC4294337

[62] Wang H, Xu J, Lazarovici P, Zheng W. Dysbindin-1 Involvement in the Etiology of Schizophrenia. Int J Mol Sci. 2017 Sep;18(10). doi: 10.3390/ijms18102044 28937620 PMC5666726

[63] Ozaita A, Puighermanal E, Maldonado R. Regulation of PI3K/Akt/GSK-3 pathway by cannabinoids in the brain. J Neurochem. 2007 Aug;102(4):1105–14. doi: 10.1111/j.1471-4159.2007.04642.x 17484726

[64] Puglielli L, Tanzi RE, Kovacs DM. Alzheimer’s disease: the cholesterol connection. Nat Neurosci. 2003 Apr;6(4):345–51. doi: 10.1038/nn0403-345 12658281

[65] Cameron BM, VanderPutten DM, Merril CR. Preliminary study of an increase of a plasma apolipoprotein E variant associated with peripheral nerve damage. A finding in patients with chronic spinal pain. Spine (Phila Pa 1976). 1995 Mar;20(5):581–90. 7604328 10.1097/00007632-199503010-00014

[66] Gordon I, Ben-Eliyahu S, Rosenne E, Sehayek E, Michaelson DM. Derangement in stress response of apolipoprotein E-deficient mice. Neurosci Lett. 1996 Mar;206(2–3):212–4. doi: 10.1016/s0304-3940(96)12470-8 8710189

[67] Miller AH, Maletic V, Raison CL. Inflammation and its discontents: the role of cytokines in the pathophysiology of major depression. Biol Psychiatry. 2009 May;65(9):732–41. doi: 10.1016/j.biopsych.2008.11.029 19150053 PMC2680424

[68] Sawicki CM, McKim DB, Wohleb ES, Jarrett BL, Reader BF, Norden DM, et al. Social defeat promotes a reactive endothelium in a brain region-dependent manner with increased expression of key adhesion molecules, selectins and chemokines associated with the recruitment of myeloid cells to the brain. Neuroscience. 2015 Aug;302:151–64. doi: 10.1016/j.neuroscience.2014.10.004 25445193 PMC4397120

[69] Huang CCY, Muszynski KJ, Bolshakov VY, Balu DT. Deletion of Dtnbp1 in mice impairs threat memory consolidation and is associated with enhanced inhibitory drive in the amygdala. Transl Psychiatry. 2019 Apr;9(1):132. doi: 10.1038/s41398-019-0465-y 30967545 PMC6456574

[70] Pletnikov Mikhail V., Waddington JL, editor. Modeling the Psychopathological Dimensions of Schizophrenia From Molecules to Behavior. ScienceDirect; 2016. 3–532 p.

[71] Berk M, Desai SY, Heyman HC, Colmenares C. Mice lacking the ski proto-oncogene have defects in neurulation, craniofacial, patterning, and skeletal muscle development. Genes Dev. 1997 Aug;11(16):2029–39. doi: 10.1101/gad.11.16.2029 9284043 PMC316447

[72] Deheuninck J, Luo K. Ski and SnoN, potent negative regulators of TGF-beta signaling. Cell Res. 2009 Jan;19(1):47–57. doi: 10.1038/cr.2008.324 19114989 PMC3103856

[73] Bartelt A, Orlando P, Mele C, Ligresti A, Toedter K, Scheja L, et al. Altered endocannabinoid signalling after a high-fat diet in Apoe -/- mice: Relevance to adipose tissue inflammation, hepatic steatosis and insulin resistance. Diabetologia. 2011;54(11):2900–10. doi: 10.1007/s00125-011-2274-6 21847582

[74] Russell JC, Kelly SE, Diane A, Wang Y, Mangat R, Novak S, et al. Rimonabant-mediated changes in intestinal lipid metabolism and improved renal vascular dysfunction in the JCR:LA-cp rat model of prediabetic metabolic syndrome. Am J Physiol Gastrointest Liver Physiol [Internet]. 2010;299(2):G507–16. Available from: http://www.ncbi.nlm.nih.gov/pubmed/20508159 doi: 10.1152/ajpgi.00173.2010 20508159

[75] Babenko VN, Smagin DA, Kudryavtseva NN. RNA-Seq Mouse Brain Regions Expression Data Analysis: Focus on ApoE Functional Network. J Integr Bioinform [Internet]. 2017;0(0). Available from: http://www.degruyter.com/view/j/jib.ahead-of-print/jib-2017-0024/jib-2017-0024.xml doi: 10.1515/jib-2017-0024 28902624 PMC6042815

[76] Josephson A, Trifunovski A, Widmer HR, Widenfalk J, Olson L, Spenger C. Nogo-receptor gene activity: cellular localization and developmental regulation of mRNA in mice and humans. J Comp Neurol. 2002 Nov;453(3):292–304. doi: 10.1002/cne.10408 12378589

[77] Mi S, Miller RH, Lee X, Scott ML, Shulag-Morskaya S, Shao Z, et al. LINGO-1 negatively regulates myelination by oligodendrocytes. Nat Neurosci. 2005;8(6):745–51. doi: 10.1038/nn1460 15895088

[78] Petratos S, Azari MF, Ozturk E, Papadopoulos R, Bernard CC a. Novel therapeutic targets for axonal degeneration in multiple sclerosis. J Neuropathol Exp Neurol [Internet]. 2010;69(4):323–34. Available from: http://www.ncbi.nlm.nih.gov/pubmed/20448478 10.1097/NEN.0b013e3181d60ddb 20448478

[79] Akbik F, Cafferty WBJ, Strittmatter SM. Myelin associated inhibitors: a link between injury-induced and experience-dependent plasticity. Exp Neurol. 2012 May;235(1):43–52. doi: 10.1016/j.expneurol.2011.06.006 21699896 PMC3189418

[80] Tomas-Roig J, Wirths O, Salinas-Riester G, Havemann-Reinecke U. The Cannabinoid CB1/CB2 Agonist WIN55212.2 Promotes Oligodendrocyte Differentiation In Vitro and Neuroprotection During the Cuprizone-Induced Central Nervous System Demyelination. CNS Neurosci Ther. 2016;22(5). doi: 10.1111/cns.12506 26842941 PMC5067581

[81] Bifulco M, Laezza C, Stingo S, Wolff J. 2’,3’-Cyclic nucleotide 3’-phosphodiesterase: a membrane-bound, microtubule-associated protein and membrane anchor for tubulin. Proc Natl Acad Sci U S A. 2002;99(4):1807–12. doi: 10.1073/pnas.042678799 11842207 PMC122275

[82] Gobert RP, Joubert L, Curchod M-L, Salvat C, Foucault I, Jorand-Lebrun C, et al. Convergent functional genomics of oligodendrocyte differentiation identifies multiple autoinhibitory signaling circuits. Mol Cell Biol. 2009;29(6):1538–53. doi: 10.1128/MCB.01375-08 19139271 PMC2648232

[83] Gravel M, Robert F, Kottis V, Gallouzi IE, Pelletier J, Braun PE. 2′,3′‐Cyclic nucleotide 3′‐phosphodiesterase: A novel RNA-binding protein that inhibits protein synthesis. J Neurosci Res. 2009;87(5):1069–79. doi: 10.1002/jnr.21939 19021295

[84] Lee J, Gravel M, Zhang R, Thibault P, Braun PE. Process outgrowth in oligodendrocytes is mediated by CNP, a novel microtubule assembly myelin protein. J Cell Biol. 2005;170(4):661–73. doi: 10.1083/jcb.200411047 16103231 PMC2171497

[85] Boggs JM. Myelin basic protein: a multifunctional protein. Cell Mol Life Sci. 2006 Sep;63(17):1945–61. doi: 10.1007/s00018-006-6094-7 16794783 PMC11136439

[86] Velasco L, Ruiz L, Sánchez MG, Díaz-Laviada I. delta(9)-Tetrahydrocannabinol increases nerve growth factor production by prostate PC-3 cells. Involvement of CB1 cannabinoid receptor and Raf-1. Eur J Biochem [Internet]. 2001;268(3):531–5. Available from: http://www.ncbi.nlm.nih.gov/pubmed/11168391 doi: 10.1046/j.1432-1327.2001.01884.x 11168391

[87] Crunelle CL, Van De Giessen E, Schulz S, Vanderschuren LJMJ, De Bruin K, Van Den Brink W, et al. Cannabinoid-1 receptor antagonist rimonabant (SR141716) increases striatal dopamine D2 receptor availability. Addict Biol. 2013;18(6):908–11. doi: 10.1111/j.1369-1600.2011.00369.x 21955259 PMC3252421

[88] Soliman N, Okuse K, Rice ASC. VGF: a biomarker and potential target for the treatment of neuropathic pain? Pain reports. 2019;4(5):e786. doi: 10.1097/PR9.0000000000000786 31875189 PMC6882576

[89] Ting S-M, Zhao X, Sun G, Obertas L, Ricote M, Aronowski J. Brain Cleanup as a Potential Target for Poststroke Recovery: The Role of RXR (Retinoic X Receptor) in Phagocytes. Stroke. 2020 Mar;51(3):958–66. doi: 10.1161/STROKEAHA.119.027315 31914884 PMC7042051

[90] Cao H, Li M-Y, Li G, Li S-J, Wen B, Lu Y, et al. Retinoid X Receptor α Regulates DHA-Dependent Spinogenesis and Functional Synapse Formation In Vivo. Cell Rep. 2020 May;31(7):107649. doi: 10.1016/j.celrep.2020.107649 32433958

[91] Huang JK, Jarjour AA, Nait Oumesmar B, Kerninon C, Williams A, Krezel W, et al. Retinoid X receptor gamma signaling accelerates CNS remyelination. Nat Neurosci. 2011;14(1):45–53. doi: 10.1038/nn.2702 21131950 PMC4013508

[92] Tomas-Roig J, Agbemenyah HY, Celarain N, Quintana E, Ramió-Torrentà L, Havemann-Reinecke U. Dose-dependent effect of cannabinoid WIN-55,212–2 on myelin repair following a demyelinating insult. Sci Rep. 2020;10(1). doi: 10.1038/s41598-019-57290-1 31953431 PMC6969154

[93] Lucchinetti CF, Brück W, Rodriguez M, Lassmann H. Distinct patterns of multiple sclerosis pathology indicates heterogeneity on pathogenesis. Brain Pathol. 1996 Jul;6(3):259–74. doi: 10.1111/j.1750-3639.1996.tb00854.x 8864283 PMC7161824

[94] Jiang C, Lin W-J, Sadahiro M, Labonté B, Menard C, Pfau ML, et al. VGF function in depression and antidepressant efficacy. Mol Psychiatry. 2018 Jul;23(7):1632–42. doi: 10.1038/mp.2017.233 29158577 PMC5962361

[95] Alves LQ, Alves J, Ribeiro R, Ruivo R, Castro F. The dopamine receptor D(5) gene shows signs of independent erosion in toothed and baleen whales. PeerJ. 2019;7:e7758. doi: 10.7717/peerj.7758 31616587 PMC6791347

[96] Yang Y, Zhang K, Zhong J, Wang J, Yu Z, Lei X, et al. Stably maintained microtubules protect dopamine neurons and alleviate depression-like behavior after intracerebral hemorrhage. Sci Rep. 2018 Aug;8(1):12647. doi: 10.1038/s41598-018-31056-7 30140021 PMC6107628

[97] Barko K, Paden W, Cahill KM, Seney ML, Logan RW. Sex-Specific Effects of Stress on Mood-Related Gene Expression. Mol neuropsychiatry. 2019 Jun;5(3):162–75. doi: 10.1159/000499105 31312637 PMC6597924

[98] Yanovich C, Kirby ML, Michaelevski I, Yadid G, Pinhasov A. Social rank-associated stress vulnerability predisposes individuals to cocaine attraction. Sci Rep. 2018 Jan;8(1):1759. doi: 10.1038/s41598-018-19816-x 29379100 PMC5789078

[99] Mukhopadhyay B, Liu J, Osei-Hyiaman D, Godlewski G, Mukhopadhyay P, Wang L, et al. Transcriptional regulation of cannabinoid receptor-1 expression in the liver by retinoic acid acting via retinoic acid receptor-gamma. J Biol Chem. 2010 Jun;285(25):19002–11. doi: 10.1074/jbc.M109.068460 20410309 PMC2885177

[100] Biswas S, Chung SH, Jiang P, Dehghan S, Deng W. Development of glial restricted human neural stem cells for oligodendrocyte differentiation in vitro and in vivo. Sci Rep. 2019 Jun;9(1):9013. doi: 10.1038/s41598-019-45247-3 31227736 PMC6588721

[101] Xapelli S, Agasse F, Grade S, Bernardino L, Ribeiro FF, Schitine CS, et al. Modulation of subventricular zone oligodendrogenesis: a role for hemopressin? Front Cell Neurosci. 2014;8(59):1–9. doi: 10.3389/fncel.2014.00059 24578683 PMC3936357

[102] Miguel-Hidalgo JJ, Carter K, Deloach PH, Sanders L, Pang Y. Glucocorticoid-Induced Reductions of Myelination and Connexin 43 in Mixed Central Nervous System Cell Cultures Are Prevented by Mifepristone. Neuroscience. 2019 Jul;411:255–69. doi: 10.1016/j.neuroscience.2019.05.050 31163207 PMC6664452

[103] Laprairie RB, Kelly MEM, Denovan-Wright EM. The dynamic nature of type 1 cannabinoid receptor (CB(1)) gene transcription. Br J Pharmacol [Internet]. 2012;167(8):1583–95. Available from: http://doi.wiley.com/10.1111/j.1476-5381.2012.02175.x%5Cnhttp://www.pubmedcentral.nih.gov/articlerender.fcgi?artid=3525862&tool=pmcentrez&rendertype=Abstract 22924606 10.1111/j.1476-5381.2012.02175.x PMC3525862

[104] Bortolato M, Mangieri RA, Fu J, Kim JH, Arguello O, Duranti A, et al. Antidepressant-like activity of the fatty acid amide hydrolase inhibitor URB597 in a rat model of chronic mild stress. Biol Psychiatry. 2007 Nov;62(10):1103–10. doi: 10.1016/j.biopsych.2006.12.001 17511970

[105] Liu X, Betzenhauser MJ, Reiken S, Meli AC, Xie W, Chen BX, et al. Role of leaky neuronal ryanodine receptors in stress- induced cognitive dysfunction. Cell. 2012;150(5):1055–67. doi: 10.1016/j.cell.2012.06.052 22939628 PMC3690518

[106] Fitzjohn SM, Collingridge GL. Calcium stores and synaptic plasticity. Cell Calcium. 2002;32(5–6):405–11. doi: 10.1016/s0143416002001999 12543099

[107] Mattson MP. Calcium and neurodegeneration. Aging Cell. 2007 Jun;6(3):337–50. doi: 10.1111/j.1474-9726.2007.00275.x 17328689

[108] Nakamura-Maruyama E, Kai R, Himi N, Okabe N, Narita K, Miyazaki T, et al. Ryanodine receptors are involved in the improvement of depression-like behaviors through electroconvulsive shock in stressed mice. Brain Stimul. 2021;14(1):36–47. doi: 10.1016/j.brs.2020.11.001 33166727

[109] Isokawa M, Alger BE. Ryanodine receptor regulates endogenous cannabinoid mobilization in the hippocampus. J Neurophysiol. 2006 May;95(5):3001–11. doi: 10.1152/jn.00975.2005 16467427

[110] Scott BL, Welch K, deSerrano V, Moss NC, Roses AD, Strittmatter WJ. Human apolipoprotein E accelerates microtubule polymerization in vitro. Neurosci Lett. 1998 Apr;245(2):105–8. doi: 10.1016/s0304-3940(98)00180-3 9605496

[111] Jiang Q, Lee CYD, Mandrekar S, Wilkinson B, Cramer P, Zelcer N, et al. ApoE promotes the proteolytic degradation of Abeta. Neuron. 2008 Jun;58(5):681–93. doi: 10.1016/j.neuron.2008.04.010 18549781 PMC2493297

[112] Balmik AA, Sonawane SK, Chinnathambi S. Modulation of Actin network and Tau phosphorylation by HDAC6 ZnF UBP domain. bioRxiv [Internet]. 2019 Jan 1;702571. Available from: http://biorxiv.org/content/early/2019/07/14/702571.abstract

