# Supplementary figures and images for: Psychosocial stress and cannabinoid drugs affect acetylation of α-tubulin (K40) and gene expression in the prefrontal cortex of adult mice
Source: PLoS One. 2022 Sep 21;17(9):e0274352. doi: 10.1371/journal.pone.0274352 (PMC9491557; doi:10.1371/journal.pone.0274352)

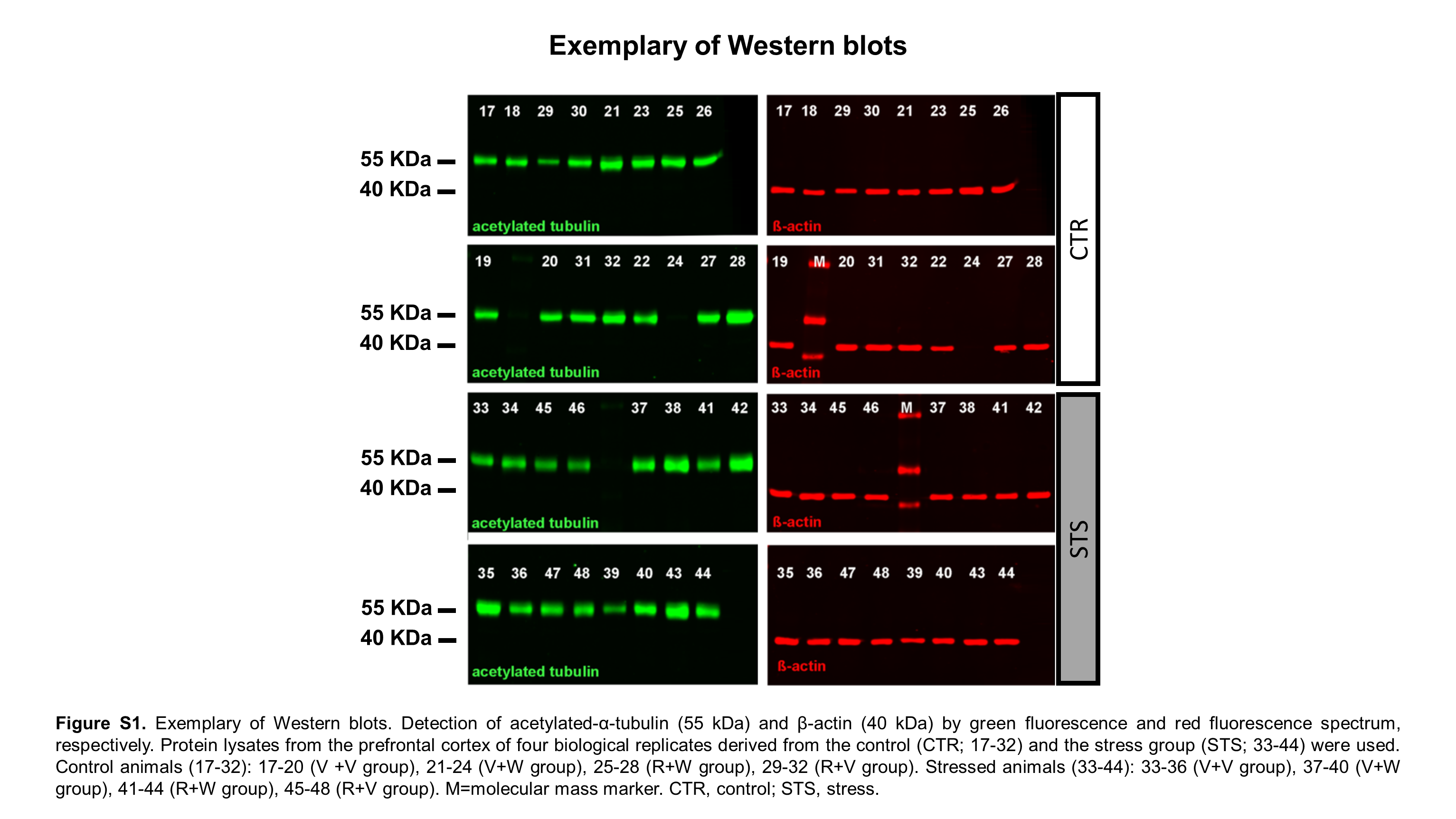

Supplement: S1 Fig — Detection of acetylated-α-tubulin (55 kDa) and β-actin (40 kDa) by green fluorescence and red fluorescence spectrum, respectively. Protein lysates from the prefrontal cortex of four biological replicates derived from the control (CTR; 17–32) and the stress group (STS; 33–44) were used. Control animals (17–32): 17–20 (V +V group), 21–24 (V+W group), 25–28 (R+W group), 29–32 (R+V group). Stressed animals (33–44): 33–36 (V+V group), 37–40 (V+W group), 41–44 (R+W group), 45–48 (R+V group). M = molecular mass marker. CTR, control; STS, stress. (TIF) [file pone.0274352.s001.tif]
