# Supplementary material for: Psychosocial stress and cannabinoid drugs affect acetylation of α-tubulin (K40) and gene expression in the prefrontal cortex of adult mice
Source: PLoS One. 2022 Sep 21;17(9):e0274352. doi: 10.1371/journal.pone.0274352 (PMC9491557; doi:10.1371/journal.pone.0274352)
Supplement: S1 File — (DOCX) [file pone.0274352.s003.docx]

**S1_raw_images
Raw images of all Western blots used for the quantification of acetylated alpha-tubulin.** All probes were loaded onto 4 gels and processed at the same time using the same antibody dilutions, washing procedures, and image capture, and replicated several times. Western blots were incubated with the indicated antibodies, anti acetylated-α-tubulin and anti ß-actin followed by fluorophore-labelled secondary antibodies, goat anti mouse IRDye800CW (green) and goat anti rabbit IRDye680RD (red). Images were captured with Odyssey CLx Imaging System and quantified using the software Image StudioTMLite (both LI-COR Biosciences, Nebraska, USA).
M=molecular mass marker (PageRuler Prestained Protein Ladder, #26616, Thermo Scientific).

Identity of probes of the prefrontal cortex (PFC)

CTR (controls): vehicle (V+V): 17, 18,19,20
 rimonabant (R+V): 29,30,31,32
 WIN55,212-2 (V+W): 21, 22, 23 24
 rimonabant and WIN55,212-2 (R+W): 25, 26, 27, 28
Psychosocial stress (STS): vehicle (V+V): 33, 34, 35, 36
 R+V: 45, 46, 47, 48
 V+W: 37, 38, 39, 40
 R+W: 41, 42, 43, 44.

Identity of probes of the dorsal striatum (DS)

CTR (controls): vehicle (V+V): 49, 50, 51 52
 rimonabant (R+V): 61, 62, 63, 64
 WIN55,212-2 (V+W): 53 54, 55, 56
 rimonabant and WIN55,212-2 (R+W): 57, 58, 59, 60
Psychosocial stress (STS): vehicle (V+V): 65, 66, 67, 68
 R+V: 77, 78, 79, 80
 V+W: 69, 70, 71, 72
 R+W: 73, 74, 75, 76.

Identity of probes of the cerebellum (CRB)

CTR (controls): vehicle (V+V): 81, 82, 83,84
 rimonabant (R+V): 93, 94, 95, 96
 WIN55,212-2 (V+W): 85, 86, 87, 88
 rimonabant and WIN55,212-2 (R+W): 89, 90, 91, 92
Psychosocial stress (STS): vehicle (V+V): 97, 98, 99, 100
 R+V: 109, 110, 111, 112
 V+W: 101, 102, 103, 104
 R+W: 105, 106, 107, 108.

Identity of probes of the hippocampus (HIPP))

CTR (controls): vehicle (V+V): 113, 114, 115, 116
 rimonabant (R+V): 125, 126, 127, 128
 WIN55,212-2 (V+W): 117, 118, 119, 120
 rimonabant and WIN55,212-2 (R+W): 121, 122, 123, 124
Psychosocial stress (STS): vehicle (V+V): 129, 130, 131, 132
 R+V: 141, 142, 143, 144
 V+W: 133, 134, 135, 136
 R+W: 137, 138, 139, 140.
